# Bilayered Two-Dimensional Hybrid Perovskite with Switchable Dielectric Properties, Persistent Disorder, and Narrow-Band Emission

**DOI:** 10.1021/acs.inorgchem.6c02525

**Published:** 2026-08-04

**Authors:** Jan Kudrawiec, Mirosław Mączka, Andrzej Nowok, Anna Gągor, Rafał Bartoszewicz, Daria Szewczyk, Adam Sieradzki

**Affiliations:** † 215275 Institute of Low Temperature and Structure Research, Polish Academy of Sciences, ul. Okólna 2, 50-422 Wrocław, Poland; ‡ Department of Experimental Physics, 49567 Wrocl̷aw University of Science and Technology, Wybrzeże Wyspiańskiego 27, 50-370 Wrocl̷aw, Poland; § Department of Semiconductor Materials Engineering, Faculty of Fundamental Problems of Technology, Wrocław University of Science and Technology, Wybrzeże Wyspiańskiego 27, 50-370 Wrocław, Poland

## Abstract

Two-dimensional (2D) multilayered lead halide perovskites have shown great potential for application in optoelectronics. Here, single crystals of 2D perovskite IPA_2_FAPb_2_Br_7_ (IPA = isopropylammonium, FA = formamidinium) were grown, and the phase transition mechanism, vibrational, dielectric, and optical properties were investigated. IPA_2_FAPb_2_Br_7_ crystallizes in the *I*4/*mmm* space group and undergoes two phase transitions at 303 and 136 K. The higher-temperature one involves only partial structural ordering and is accompanied by a distinct dielectric anomaly, enabling dielectric switching. In contrast, the low-temperature transition has mainly displacive character, and Arrhenius extrapolation suggests that FA^+^ dynamics may persist down to approximately 70–80 K. Consequently, IPA_2_FAPb_2_Br_7_ acts as a dielectric switch with persistent disorder, which allows the compound to relax even at low temperatures and enables continuous modulation of its optoelectronic properties. Photoluminescent (PL) studies show that IPA_2_FAPb_2_Br_7_ exhibits narrowband emission with a maximum near 435 nm that was tentatively assigned to localized excitons, as its temperature dependence does not follow the bandgap evolution. Overall, these findings identify IPA_2_FAPb_2_Br_7_ as a promising light-emitting material and dielectric switch and highlight the importance of complementary temperature-dependent PL and transmission studies for understanding the optoelectronic properties of multilayered perovskites and the origin of PL.

## Graphical abstract

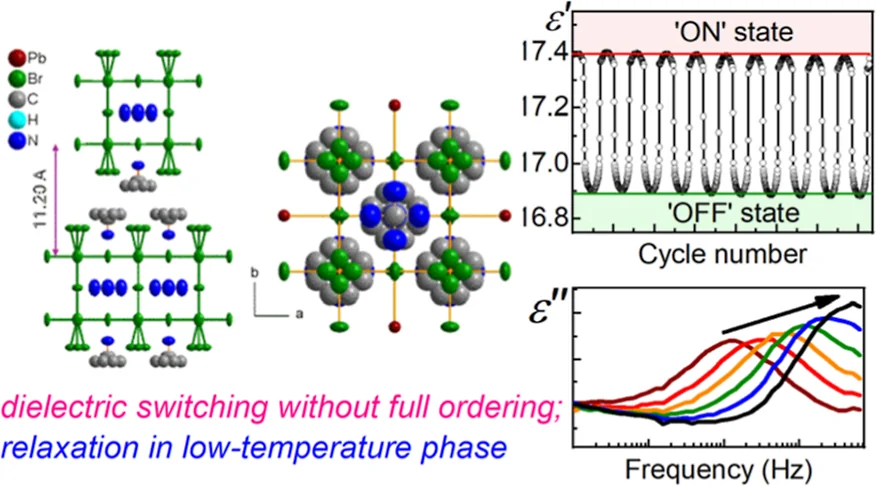

## Introduction

Hybrid organic–inorganic lead halide perovskites have received much attention in recent years due to their photovoltaic performance as well as interesting optical and electric properties.^1–4^ Important aspect of these compounds is their ability to crystallize in various dimensionalities including zero- and one-dimensional structures with different stoichiometries, layered two-dimensional (2D) structures with general formulas A′_2_PbX_4_ or A″PbX_4_, and three-dimensional (3D) phases with APbX_3_ stoichiometry (A, A′, A″, and X denote small organic monovalent cation, large organic monovalent cation metal cation, large organic divalent cation, and halogen anion, respectively). Achieving the right dimensionality allows for tuning physicochemical properties of lead halide perovskites.^1,2,4–7^ For instance, a decrease of dimensionality leads to a widening of the bandgap and an increase of the exciton binding energy, resulting in the decrease of the photovoltaic performance, but in many cases, an increase of the photoluminescence (PL) quantum yield.^1–8^ The change of dimensionality also leads to strong tunability of the emission color and may also lead to a change of the PL character from narrowband, related to the recombination of free excitons (FEs), to broadband, attributed to self-trapped excitons (STEs).^1,2,5–7^ A decrease of dimensionality is also an efficient strategy for inducing ferroelectricity, antiferroelectricity, and second-order nonlinear optical (NLO) properties (e.g., second-harmonic generation (SHG)).^9–14^ Structural diversity of 2D layered lead halide perovskites can be further increased by the employment of two different cations. Two major subgroups, as known, i.e., Dion-Jacobson (DJ) and Ruddlesden–Popper (RP) phases with general formulas A″A_*n*–1_Pb_*n*_X_3*n*–1_ and A′_2_A_*n*–1_Pb_*n*_X_3*n*–1_, respectively, where *n* indicates the number of octahedral layers coupled together via apical X^–^ anions and small A^+^ cations occupy intralayer sites (perovskite cages) and large A′^+^ or A″^2+^ cations separate the perovskite layers.^1,7,8,12^ DJ structures are known to have enhanced environmental stability and shorter interlayer distance compared with RP analogues due to the replacement of van der Waals interactions by much stronger hydrogen-bond (HB) interactions.^15,16^ Employment of various inter- and intralayer cations and their ratio allows control of crystal symmetry and structural parameters (*n*, octahedral distortion, etc.) of multilayered perovskites and, in turn, their properties.^8,12,15–17^ In particular, an increase of *n* leads to narrowing of the bandgap and a decrease of the exciton binding energy, which in turn improves photovoltaic performance and results in a red shift of the PL.^7,8^ Therefore, changing the *n* is one of the factors that allows tuning the optoelectronic properties of multilayered perovskites in search of next-generation optoelectronic devices.^7,8,18,19^ It is worth noting that organic cations in multilayered perovskites are often disordered at elevated temperatures but ordered with decreasing temperature. This ordering may occur at the same temperature for both cations or at two different temperatures (ordering of the interlayer cations at a higher temperature followed by ordering of the cage cations at a lower temperature or vice versa) or in a gradual way.^13,14,20–25^ Very recently, Monte Carlo simulations showed that the type of ordering and presence of one or two phase transitions (PTs) depend on the magnitude of short-range framework-mediated interactions between molecular cations.^26^ Most importantly, the ordering of organic cations and the flexibility of RP phases allow for symmetry-breaking at the PTs, often leading to noncentrosymmetric structures.^9,11,12,20,21,23,25,27^ This, in turn, leads to the appearance of technologically important NLO properties and/or ferro-, pyro-, and piezoelectricity.^9,11,12,20,21,23,25,27^ Furthermore, the change of organic cation dynamics at a PT may lead to a change of the dielectric permittivity from a low (static) to high (dynamic) state. This dielectric switching phenomenon received great attention in recent years since switchable dielectrics are attractive materials for energy storage, switching, sensing, and memory devices.^28–30^

Regarding the A^+^ cation, its size, shape, and ability to form HBs strongly have significant effects on octahedral distortion and molecular dynamics and, in turn, on optoelectronic and electrical properties. In this regard, the formamidinium cation ((CH­(NH_2_)_2_)^+^, FA^+^) has emerged as an alternative for the widely used methylammonium cation (CH_3_NH_3_^+^, MA^+^) due to its higher ability to form HBs, which leads to enhanced stability of the perovskites.^31,32^ Multilayered lead bromide perovskites comprising FA^+^ cations have been reported for a number of interlayer cations such as butylammonium (BA^+^), isobutylammonium (IBA^+^), propylammonium (PA^+^), 3-bromopropylammonium (BPA^+^), and 3-chloropropylammonium (CPA^+^).^12,20–23^ Interestingly, in contrast to MA^+^ and ethylammonium (EA^+^) analogues, which form both *n* = 2 and *n* = 3 phases, only bilayered (*n* = 2) analogues are known for FA^+^ counterparts.^12,20–23^ The available data show that the majority of these bromides exhibit ferroelectric properties and that changing of the interlayer cation allows tuning of the PT temperature from 263.3 K for PA^+^ to 348.5 K for BPA^+^.^12,20,21,23^ Apart from bromides, iodides are also known, such as BA_2_FAPb_2_I_7_ and IPA_2_FAPb_2_I_7_ (IPA = isopropylammonium).^27,32,33^ The former one exhibits ferroelectric and photodetection properties, while the latter one was reported to exhibit a giant entropy change (∼40 Jmol^–1^K^–1^) and superior PL.^27,33^ It should be noted that studies of the FA-based bilayered perovskites focused mainly on their ferro- or antiferroelectric and photodetection properties.^12,20–23,27^ Detailed analysis of their dielectric properties is missing, and temperature-dependent PL studies were reported for the IPA_2_FAPb_2_I_7_ iodide only.^33^ Furthermore, reported room-temperature PL data for FA-based bilayered perovskites are not consistent with the absorption data, showing a strongly Stokes-shifted narrow band near 526–527 nm, suggesting its origin from 3D FAPbBr_3_ impurity rather than RP phases.^20,21^

One of the important parameters considered in engineering 2D structures is the minimization of interlayer distance separating the inorganic layers and organic layers acting as a dielectric barrier hindering charge transport in the crystal.^8,34^ This can be achieved by using a small interlayer cation, and one of the possible choices is isopropylammonium (IPA^+^). Indeed, it has been reported that IPA_2_MAPb_2_I_7_, which crystallizes in a centrosymmetric tetragonal structure, exhibits a very short interlayer distance of 5.15 Å.^35^ Among bromides, six compounds have been reported, i.e., antiferroelectric IPA_2_CsPb_2_Br_7_,^36^ IPA_2_Tz_*n*–1_Pb_*n*_Br_3*n*+1_ (*n* = 2,3), which are the only representatives of multilayered perovskites comprising large 1,2,4-triazolium (Tz^+^) cations in the intralayer perovskite sites,^37^ IPA_2_MA_2_Pb_3_Br_3_, exhibiting SHG activity below 253 K,^38^ IPA_2_DMAPb_2_Br_7_ (DMA = dimethylammonium),^38^ and IPA_2_(IPA_0.77_MHy_0.23_)­Pb_2_Br_7_ (MHy = methylhydrazinium).^38^ The latter compound is the first example reporting that IPA^+^ may be located in both the interlayer space and inside perovskite cavities.^38^

It should be added that despite the interesting optoelectronic properties of lead halide perovskites, the presence of lead remains one of the major concerns associated with technological implementation of such materials because lead belongs to the family of toxic metals.^39^ However, replacing Pb while preserving the favorable electronic structure, the optical responses, photovoltaic efficiency, and defect-tolerant character of lead perovskites remains a major challenge.^40^ Therefore, the Pb-based systems remain essential systems for fundamental studies, as their remarkable properties provide a crucial reference point for understanding structure–property relationships in hybrid perovskites and allow for guidance in the rational design of safer lead-free alternatives.

This short survey shows that multilayered perovskites comprising FA^+^ cations have great potential for applications such as nonlinear optical (NLO), ferroelectric, antiferroelectric, and PL materials. However, detailed knowledge of their dielectric and optical properties in a broad temperature range is still lacking. In this respect, dielectric studies are important for better understanding the mechanism of the PTs and the suitability of a perovskite as a switchable dielectric, ferroelectric, or antiferroelectric material. On the other hand, temperature-dependent optical studies provide important information about exciton binding energy or energy transfer between different states (for instance, between FEs and STEs). Literature data also show that employment of small IPA^+^ as an interlayer cation is an efficient way for the synthesis of layered compounds with very small interlayer distances. However, the number of reported IPA-based mixed-cation lead halides remains relatively small (seven such compounds are known). To fill this gap, we have undertaken the synthesis of a new bilayered perovskite by combining both IPA^+^ and FA^+^ cations. We have successfully synthesized IPA_2_FAPb_2_Br_7_ with a short interlayer distance and a combined multitechnique approach (single-crystal X-ray diffraction, differential scanning calorimetry (DSC), Raman, infrared (IR), optical absorption, PL, and dielectric spectroscopy) to fully describe its structural, phonon, optical, and dielectric properties.

## Experimental Section

### Materials and Crystal Growth

All reagents (PbBr_2_ 98%, HBr 48 wt % in H_2_O, formamidine acetate (99%)) and isopropylamine (99.5%) used for the synthesis were commercially purchased from Sigma-Aldrich and used without further purification. In order to grow single crystals of IPA_2_FAPb_2_Br_7_, 6 mmol of PbBr_2_ was dissolved in HBr at 50 °C under stirring on a hot plate. Then, 6 mmol of isopropylamine and 2 mmol of formamidine acetate were added. The heating was switched off, and the solution was left undisturbed at 30 °C. The yellow plate-like crystals were separated from the mother liquid after 2 days and dried. Photos of the grown crystals are shown in Figure S1. The purity of the bulk sample was confirmed by the powder X-ray diffraction (Figure S2).

### X-ray Powder Diffraction

Powder XRD patterns were measured in the reflection mode on an X’Pert PRO X-ray diffraction system equipped with a PIXcel ultrafast line detector and Soller slits for CuKα_1_ radiation (λ = 1.54056 Å).

### Differential Scanning Calorimetry (DSC)

DSC measurements were performed by using a Mettler Toledo DSC-3 calorimeter. The experiment was conducted under a nitrogen purge (30 mL min^–1^) using a 23.81 mg sample sealed in an aluminum pan. Thermograms were collected between 115 and 320 K during both heating and cooling at a rate of 2 K min^–1^. The excess heat capacity related to the PT was estimated by subtracting the baseline from the acquired data.

### Heat Capacity

The heat capacity of IPA_2_FAPb_2_Br_7_ was measured using the thermal relaxation method implemented in the heat capacity option of a commercial physical property measurement system (PPMS). The measurements were performed in two separate experimental runs, as a single continuous measurement was not possible because of insufficient thermal coupling between the sample and the measurement platform, which was affected by the properties of the used thermal grease. Therefore, two different greases were used: one suitable for the 3–275 K range and another suitable above 250 K. In both runs, the measured sample consisted of several small single crystals to ensure an efficient thermal response, with total masses of 6.90 mg (first run) and 5.32 mg (second run).

### Crystal Structure

Single-crystal X-ray diffraction data were collected on a four-circle Xcalibur Atlas diffractometer (Rigaku) using Mo Kα radiation. Data collection and refinement were carried out with CrysAlisPro. An empirical absorption correction using spherical harmonics was applied with ABSPACK, as implemented in *CrysAlisPro*. The crystal structures were solved in three polymorphic phases numbered from **I** to **III** with decreasing temperature in Olex2 1.5 using SHELXT and refined with SHELXL programs.^41–43^ The structure was determined for three polymorphic phases. The main crystallographic parameters are as follows: **Phase I** (HT): tetragonal, *I*4/*mmm*, a = 6.0173(4), c = 34.761(6) Å, V = 1258.6(3) Å^3^, Z = 2, *R*_1_ = 0.047, w*R*_2_ = 0.134; **Phase II**: monoclinic, *P*2_1_/*c*, *a* = 17.246(2), *b* = 8.5597(9), *c* = 16.963 (2) Å, β = 103.82(1)°, *V* = 2431.7(5) Å^3^, *Z* = 4, *R*_1_ = 0.057, w*R*_2_ = 0.156; **Phase III**: monoclinic, *P*2_1_/*c, a* = 17.095(2), *b* = 8.4820(9), *c* = 16.931(2) Å, β = 104.22(1)°, *V* = 2379.7(5) Å^3^, *R*_1_ = 0.055, w*R*_2_ = 0.138. Due to pronounced disorder in the high-temperature (HT) phase, hydrogen atoms were not included in the refinement. Both monoclinic phases were refined as nonmerohedral twins consisting of two domains, with a BASF parameter of approximately 0.3. Table S1 summarizes the crystal data, data collection parameters, and refinement details. Pb–Br distances are listed in Table S2.

### Broadband Dielectric Spectroscopy

The dielectric properties of IPA_2_FAPb_2_Br_7_ were investigated with a Novocontrol Alpha impedance analyzer. Temperature regulation and stabilization with a precision of 0.2 K were provided by a Novocontrol Quatro system coupled to a nitrogen-gas cryostat. Temperature- and frequency-dependent measurements were performed on a single crystal between 116 and 350 K, employing probing frequencies from 10 Hz to 1 MHz. Prior to measurements, the parallel planes of the crystalline sample were coated with silver paste to ensure a high-quality electric contact. The electrodes were placed perpendicular to the [001] direction. Dielectric spectra were recorded every 2K under quasi-static conditions, i.e., after at least 60 s of thermal stabilization at each temperature point.

The stability of the dielectric switching phenomenon was further examined in the 280–320 K range over 10 temperature cycles. Each cycle included 10 min stabilization periods at the target temperature (280 or 320 K), separated by heating and cooling ramps of 4 K min^–1^. Dielectric measurements were performed every 5 s using the probing frequencies of 1, 10, and 100 kHz.

### Raman and IR Spectroscopy

The RT Raman spectrum was measured using a Bruker FT MultiRam spectrometer with YAG: Nd laser excitation at 1064 nm, and the power was set to 500 mW. The spectral resolution was 2 cm^–1^, and the measurement comprised 256 scans. Temperature-dependent Raman spectra were measured using a Renishaw inVia Raman spectrometer equipped with a confocal DM2500 Leica optical microscope, an Eclipse filter, and a CCD detector with a spectral resolution of 2 cm^–1^, averaging 3 scans per temperature point. The excitation was done using a diode laser (λ_exc_ = 830 nm). The spectrometer was equipped with a 1800 L/mm diffraction grating, and the laser power put on the cryostat was 1 mW. The temperature of the samples was controlled using either a THMS600 temperature control stage (Linkam) or a closed cycle cryostat. Temperature-dependent IR spectra were recorded in the 7–320 K range using a Nicolet iS50 FT-IR spectrometer and closed-cycle cryostat CS202AE-DMX-1AL (Advanced Research Systems) equipped with thallium bromoiodide (KRS-5) windows. The samples were prepared using the standard KBr pellet method. Each temperature measurement was comprised of 128 scans. Since this cryostat does not allow recording IR spectra at high temperatures, IR spectra in the 330–360 K range were measured using a Nicolet iN10 IR microscope equipped with a liquid nitrogen-cooled mercury–cadmium-telluride detector, a permanently aligned 15× objective, and a 0.7 numerical aperture with a working distance of 16 mm. Each measurement was comprised of 128 scans. The temperature was changed using the ZnSe Linkam cryostat cell THMS600. The spectral resolution of IR spectra was 2 cm^–1^.

### Linear Optical Studies

The room-temperature (RT) diffuse reflectance spectrum of the powdered sample was measured using the Varian Cary 5E UV–vis–NIR spectrophotometer. To measure temperature-dependent light transmission (T) through the crystal, a halogen lamp was used and passed through a monochromator (Zolix – Omni λ 300 (i)), and then monochromatic light passed through the sample placed in the cryostat. The transmitted light was detected using a silicon diode. The measured spectrum was divided by a reference spectrum, i.e., the spectrum measured without the sample.

For PL measurements, the crystal was excited with a 325 nm line from a He–Cd laser (Kimmon – IK3501R-G) with a power of 30 μW, and the laser beam was focused on the sample to a diameter of ∼200 μm. The PL signal was collected by lens optics on optical fiber and detected with a Peltier-cooled Avantes Si CCD spectrometer dedicated to the IR-VIS-UV range. Each spectrum was collected with an integration time of 4 s and averaged over three accumulations. Temperature of the sample in the T and PL measurements was controlled using a helium closed-circled cryostat (Janis – Model 350 CP).

## Results and Discussion

### DSC and C_p_

The DSC curves for IPA_2_FAPb_2_Br_7_ show a reversible heat anomaly at *T*_1_ = 303.2 K (297.8 K) during the heating (cooling) cycle (Figure S3). Significant thermal hysteresis, symmetric shape of ΔC_p_ peaks, and discontinuous change of Δ*S* and Δ*H* (Figure 1a–c) indicate the first-order character of the PT from the HT phase **I** to the intermediate phase **II**. The associated changes in ΔΗ and Δ*S* are 3.0–3.4 kJ·mol^–1^ and 10.4–11.2 J·mol^–1^K^–1^, respectively. Based on the Boltzmann equation, Δ*S* = *R* ln­(*N*), where Δ*S* is average entropy change, *R* is the gas constant, and *N* denotes the ratio of the number of distinguishable orientations, the value of. *N* is estimated to be roughly 3.5–3.8. The large value of *N* indicates that this PT has an order–disorder character. Apart from this transformation, IPA_2_FAPb_2_Br_7_ experiences an additional PT to the LT phase **III** at *T*_2_ = 136 K, which is manifested on the thermograms as a reversible weak thermal effect. Both PTs were also confirmed by additional C_p_ measurements (Figure 2). These measurements show that in addition to a strong C_p_ anomaly at 303 K, another much weaker peak appears at 136 K, which indicates the PT from phase **II** to phase **III**. Such a weak anomaly suggests that the LT PT has predominantly displacive character with a weak order–disorder contribution. Below this temperature, no other thermal effects related to structural transformations were detected, indicating phase **III** as the low-temperature (LT) phase of IPA_2_FAPb_2_Br_7_.

**Figure 1:**
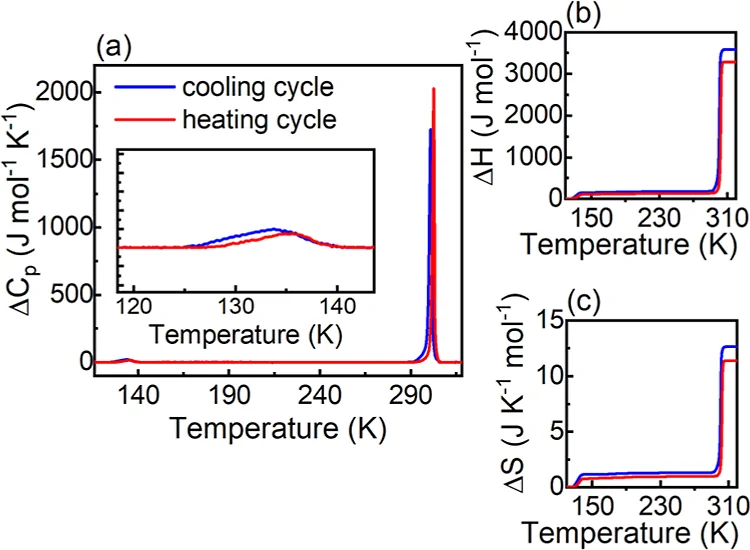
Temperature dependence of (a) excess heat capacity, (b) enthalpy, and (c) entropy for IPA_2_FAPb_2_Br_7_.

**Figure 2:**
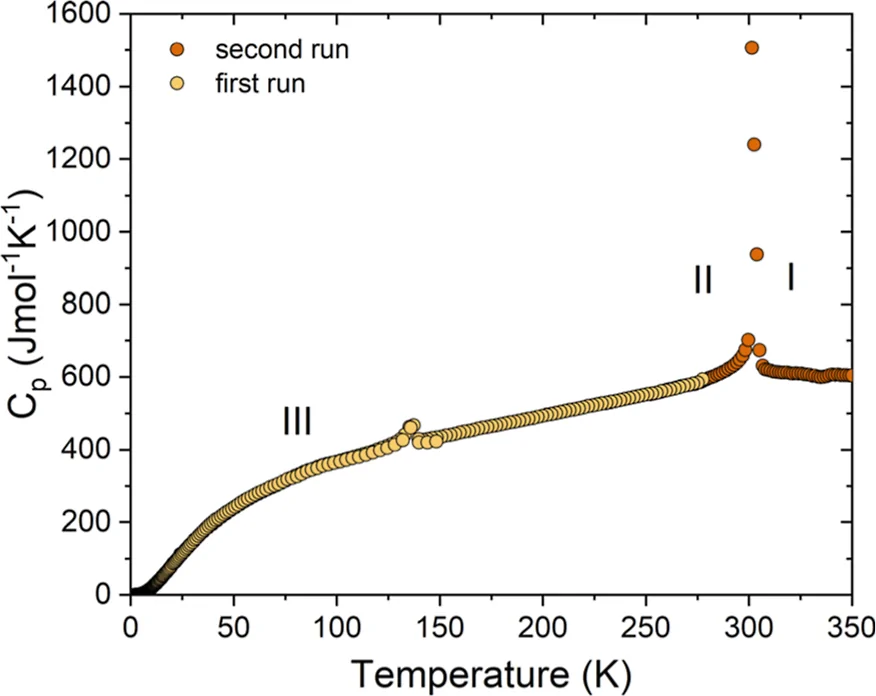
Temperature dependence of heat capacity for IPA_2_FAPb_2_Br_7_.

### Crystal Structure

IPA_2_FAPb_2_Br_7_ crystallizes in the tetragonal *I*4/*mmm* phase, forming a bilayered structure composed of corner-sharing PbBr_6_ octahedra separated by two layers of organic IPA^+^ cations connected by weak van der Waals interactions, as observed for related disordered 2D perovskites.^20–22^ Thus, IPA_2_FAPb_2_Br_7_ has a layered structure with weak interactions, allowing exfoliation. The HT phase exhibits pronounced disorder of both interlayer IPA^+^ and FA^+^ perovskitizers, which are distributed over symmetry-related positions associated with the 4-fold axis. This behavior is indicative of dynamic (time-averaged) disorder. Interactions at the organic–inorganic interface are relatively weak, as reflected by the splitting of terminal, nonbonding Br positions. Consistently, all atoms display large anisotropic displacement parameters (ADPs), suggesting significant thermal motion and enhanced rotational freedom of the molecular cations. The overall structure of phase **I** is therefore dominated by temperature-driven disorder (Figure 3a–c).

**Figure 3:**
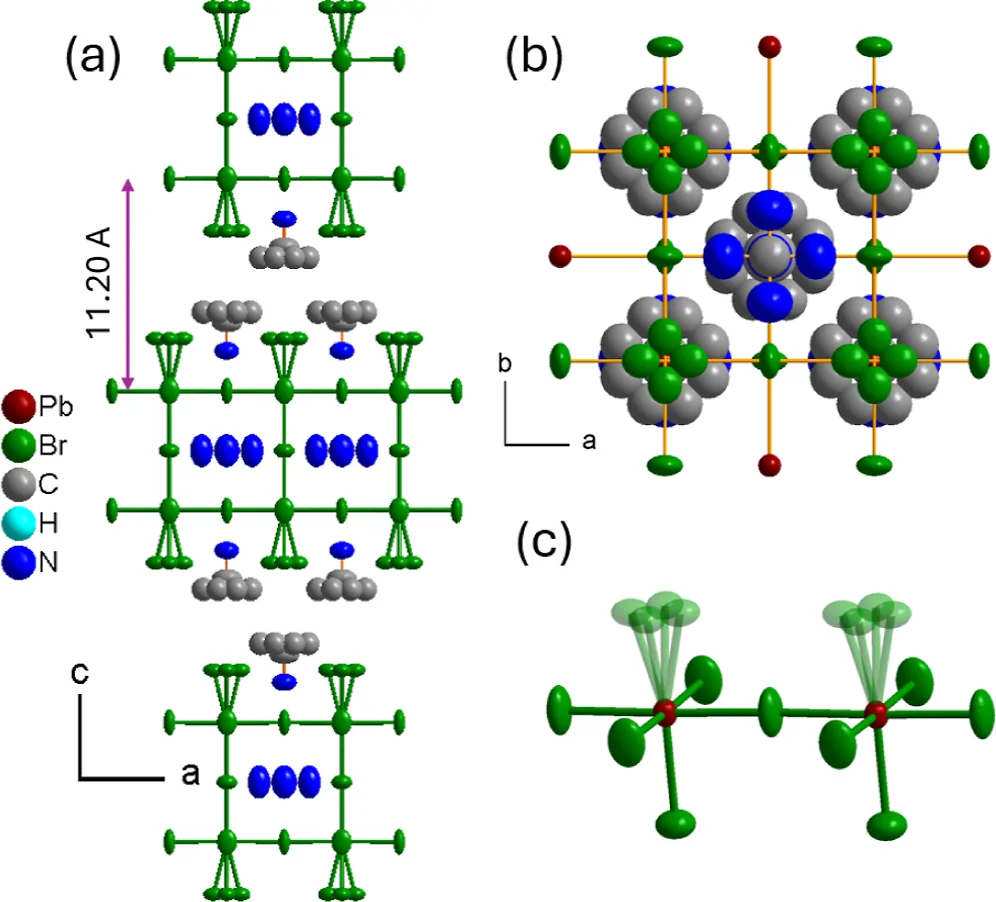
(a) Crystal structure of IPA_2_FAPb_2_Br_7_ in the HT, tetragonal phase **I** at *T* = 330 K. (b) Organic moieties disordered about the tetragonal 4-fold axis. (c) Splitting of Br atoms at the organic–inorganic interface over four equivalent positions.

Upon cooling, a PT at *T*_1_ ≈ 300 K leads to symmetry lowering from tetragonal *I*4/*mmm* to monoclinic *P*2_1_/*c*, accompanied by the formation of a domain structure. At RT, the primary diffraction patterns can be indexed in a pseudotetragonal unit cell; additional reflections from monoclinic twin domains are too weak to permit reliable structure refinement near the PT temperature (Figure S4). PT is also associated with a significant contraction of the interlayer spacing: the distance between nonbonding Br atoms from adjacent layers decreases from 5.64 to 5.05 Å, while the separation between Pb layers is reduced from 11.20 to 10.48 Å, indicating a pronounced densification of the layered framework.

Well-resolved diffraction data suitable for full structural analysis were obtained at 200 K (phase **II**) and 95 K (phase **III**). Both LT phases are isostructural, adopting the *P*2_1_/*c* space group. They are characterized by ordered, yet significantly distorted perovskite bilayers. In phase **II**, the FA^+^ cations show partial disorder, while in phase **III**, these cations are ordered at 95 K and anchored within the inorganic framework via N–H···Br HBs.

The interlayer IPA^+^ cations exhibit positional disorder in both phases, manifested by split atomic sites. The nearly equal site-occupancy factors observed for these positions indicate a static character of this disorder. The crystal structure at 95 K (Figure 4) shows pronounced tilting and distortion of the PbBr_6_ octahedra. The asymmetric unit contains a Pb_2_Br_7_ moiety with octahedral distortion parameters Δ and angle variance σ^2^ of 0.028° and 31°^2^ for Pb1, and 0.021° and 24°^2^ for Pb2, respectively. These values remain nearly unchanged across the **II**–**III** transition (200 K: Δ = 0.026 and σ^2^ = 26°^2^ for Pb1; Δ = 0.020 and σ^2^ = 22.7°^2^ for Pb2), indicating that the local distortion of the inorganic framework is largely preserved.

**Figure 4:**
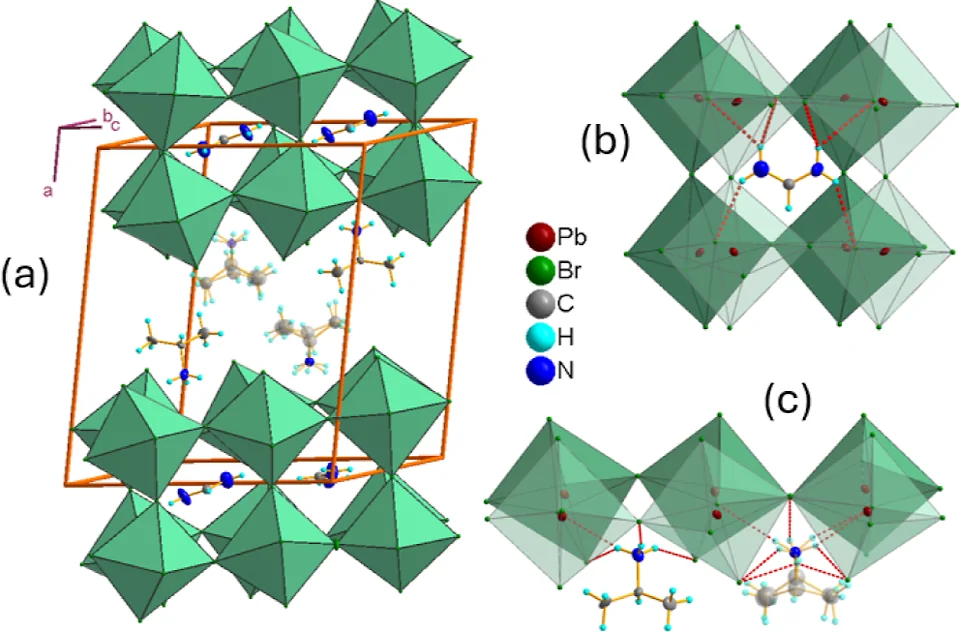
(a) Crystal structure of IPA_2_FAPb_2_Br_7_ in the LT ordered monoclinic phase **III** at *T* = 95 K. (b) FA^+^ anchored within the perovskite cavity by HBs, red dashed lines. (c) N–H···Br HBs at the organic–inorganic interface. Disorder of IPA^+^ is evidenced by split, partially transparent atomic positions.

The octahedral distortion is primarily influenced by N–H···Br hydrogen bonding in phases **II** and **III**, involving NH_2_ groups of FA^+^ and NH_3_ donors of IPA^+^ interacting with Br acceptors. A detailed description of the HB scheme, including geometrical parameters and atom numbering, is provided in Table S3 and Figure S5 in the Supporting Information.

### IR and Raman Studies

To obtain further information on the effect of PTs on lattice dynamics and the mechanism of these transitions, we have performed temperature-dependent Raman and IR studies of IPA_2_FAPb_2_Br_7_. Since temperature-dependent Raman bands are very weak in the internal modes’ region, to facilitate assignment of modes, we have also measured the RT Raman spectrum using an FT-Raman spectrometer. The spectra are presented in Figures 5 and S6–S11. Tables S4 and S5 list the observed wavenumbers and proposed assignment of the observed modes based on previous studies of FAPbBr_3_ and IPA-related compounds.^44–47^

**Figure 5:**
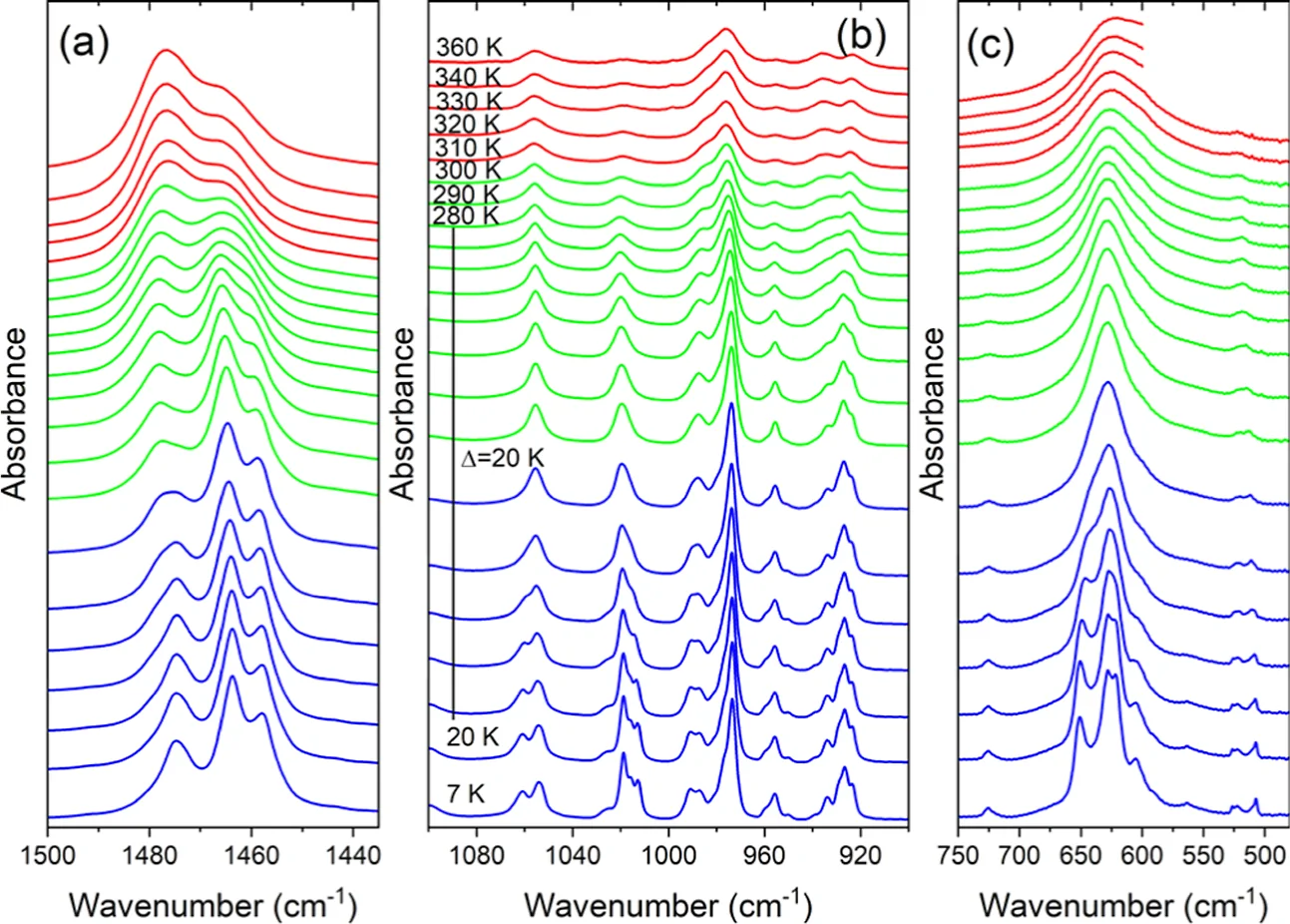
Temperature-dependent IR spectra of IPA_2_FAPb_2_Br_7_ in (a) 1500–1430 cm^–1^, (b) 1080–900 cm^–1^, and (c) 750–480 cm^–1^ for IPA_2_FAPb_2_Br_7_. The spectra in the 7–320 K were acquired using a cryostat, and those at 330, 340, and 360 K using a stand-alone IR microscope, which allows measurements down to 600 cm^–1^.

The Raman spectrum in the lattice modes region recorded at 360 K shows the presence of very broad bands at 31 and 48 cm^–1^, which can be attributed to the L­(PbBr_6_) and Pb–Br bend modes, respectively (Figure S7 and Table S4). The very large width of these bands is consistent with long-range disorder, in agreement with X-ray diffraction data. The **I**→**II** PT is observed as sudden shifts of the L­(PbBr_6_) and Pb–Br bend modes to 34 and 44 cm^–1^, respectively (values at 300 K) and their splitting into doublets at lower temperatures (Figure S7 and Table S4). Such pronounced shifts and splitting indicate that the PT is associated with a significant change in the PbBr_6_ tilts and lowering of crystal symmetry. Another important change is the narrowing of the lattice bands, which increases with further lowering of the temperature of phase **II** due to the decrease of the phonon–phonon anharmonic interactions. The width of these bands at 140 K is much smaller than the width of the corresponding modes of 3D FAPbBr_3_ at 100 K.^40^ This behavior proves that the PT is associated with significant ordering of organic cations and that phase **II** of 2D IPA_2_FAPb_2_Br_7_ shows less pronounced disorder and organic cation dynamics than the LT phase of 3D FAPbBr_3_. The **II**→**III** PT is clearly observed as a sudden shift of the Pb–Br bend mode from 47 cm^–1^ at 140 K to 50 cm^–1^ at 120 K (Figures S7 and S8b). This shift is, however, weak. Furthermore, the number of observed lattice modes remains almost constant, and the width of Raman bands changes weakly with decreasing temperature below 120 K. These changes indicate that the **II**→**III** PT has mainly a displacive character associated with weak changes in the PbBr_6_ tilts.

Raman spectra recorded in the internal modes region also show pronounced narrowing in phase **II** (Figures S8a and S9, see, for instance, the δ_as_(NH_3_) mode of IPA^+^ near 1580 cm^–1^). The **II**→**III** PT leads to weak changes in band widths. The only exception is the δ­(NCN) mode of FA^+^, which appears as a singlet at 519 cm^–1^ in phase **II** (140 K) but splits into two narrower components in phase **III** (Figure S8a and Table S4). Another interesting feature is the appearance of the FA-cage mode near 280 cm^–1^ in phase **III** (Figure S9). These features confirm an order–disorder character of the **I**→**II** PT. They also suggest that FA^+^ cations exhibit large vibrational motions in phase **II**, which exhibit large slowing down in phase **III**. Overall, Raman data are consistent with an order–disorder character and symmetry lowering due to **I**→**II** PT, partial dynamic disorder of FA^+^ in phase **II** and weak changes in the octahedral distortion parameters at the **II**→**III** PT established by X-ray diffraction studies.

Since, due to the weak intensity of Raman bands in the internal modes region, it is difficult to follow their temperature dependence and thus the molecular dynamics and mechanism of the PTs, we have performed additional IR studies in the 320–7 K range. IR spectra do not show any sudden narrowing of bands near 300 K, where the **I**→**II** PT occurs (Figures 5, S10 and S11). A similar behavior was reported for (BDA)­(EA)_2_Pb_3_Br_10_ at 410 K (BDA^2+^ = 1,4-butanediammonium, EA^+^ = ethylammonium), where a strong C_p_ anomaly indicated PT from a partially ordered to disordered phase.^43^ Similarly to (BDA)­(EA)_2_Pb_3_Br_10_, insensitivity of the IR-active internal modes to the **I**→**II** PT can be attributed to very strong contributions to the broadening arising from phonon–phonon anharmonic interactions, which obscure the additional contribution due to disordering of organic cations.^48^ However, on further lowering of temperature to 140 K, clear narrowing and changes in relative intensities of the IR bands are observed. It is worth noting that the narrowing is more pronounced for the IPA^+^ than FA^+^ cations (see, for instance, the ν­(CN) and τ­(NH_2_) modes of FA^+^ at 1056 and 625 cm^–1^, respectively, and the ν­(CN)^+^ ρ­(CH_3_) modes of IPA^+^ at 984, 955, and 935 cm^–1^, Figure 5b,c). This behavior indicates that the **I**→**II** PT is associated with more pronounced ordering and decrease of the motional freedom of IPA^+^ than FA^+^. Another interesting observation is that many modes of IPA^+^ exhibit splitting into 2–3 components when going from 320 to 140 K (Figures 5a,b, S10, S11, and Table S5), in line with the symmetry decrease and increase of the number of crystallographically independent IPA^+^ cations from one in phase **I** to two in phase **II**.

The **II**→**III** PT leads to further narrowing of IR bands, especially pronounced for the FA-related bands (see for instance the δ­(NH_2_), ρ­(NH_2_), ν­(CN), and τ­(NH_2_) bands near 1600, 1300, 1060–1000, and 650–600 cm^–1^, Figures 5b,c and S11). This behavior suggests that the **II**→**III** PT is associated with the final ordering of FA^+^ cations. It is worth noting, however, that the above-mentioned narrowing is still rather weak at 120 and 100 K but becomes pronounced below 80 K. Such behavior indicates that FA^+^ cations retain significant dynamics even in phase **III,** and this dynamic freezes completely below 80 K. Furthermore, many bands show additional splitting in phase **III** (Figures 5 and S11 and Table S5). According to the X-ray diffraction, this PT does not alter the crystal symmetry and the number of crystallographically independent IPA^+^ and FA^+^ cations (2 and 1, respectively). Thus, based on the structural monoclinic model and assuming significant Davydov splitting into A_u_ + B_u_ modes, the largest number of the split components should be 4 and 2 for IPA^+^ and FA^+^, respectively. The observation of up to four split components for IPA-related modes is consistent with the monoclinic model. However, significant splitting of some FA-based bands into up to four components at 7 K (see Figures 5, S11 and Table S5) suggests that the real symmetry of the **III** could be lower than the *P*2_1_/*c* symmetry solved at 95 K using the X-ray diffraction method.

### BDS Studies

The PTs are also reflected in the dielectric response of IPA_2_FAPb_2_Br_7_, as observed in both the real and imaginary parts of the complex dielectric permittivity. The temperature dependence of ε′ (Figure 6a), measured during cooling at probing frequencies between 10 Hz and 1 MHz, shows pronounced dispersion above 302 K, with ε′ decreasing systematically with increasing frequency. The dispersion markedly weakens below 302 K, i.e., after PT to the intermediate phase **II**. Additionally, a step-like anomaly in ε′ is detected near 302 K at all frequencies, providing a clear dielectric signature of the PT. At 1 MHz the change in ε′ is small (about 0.5, from 17.0 to 17.5), but the magnitude of the shift increases substantially at lower frequencies, reaching approximately 3 at 10 Hz.

**Figure 6:**
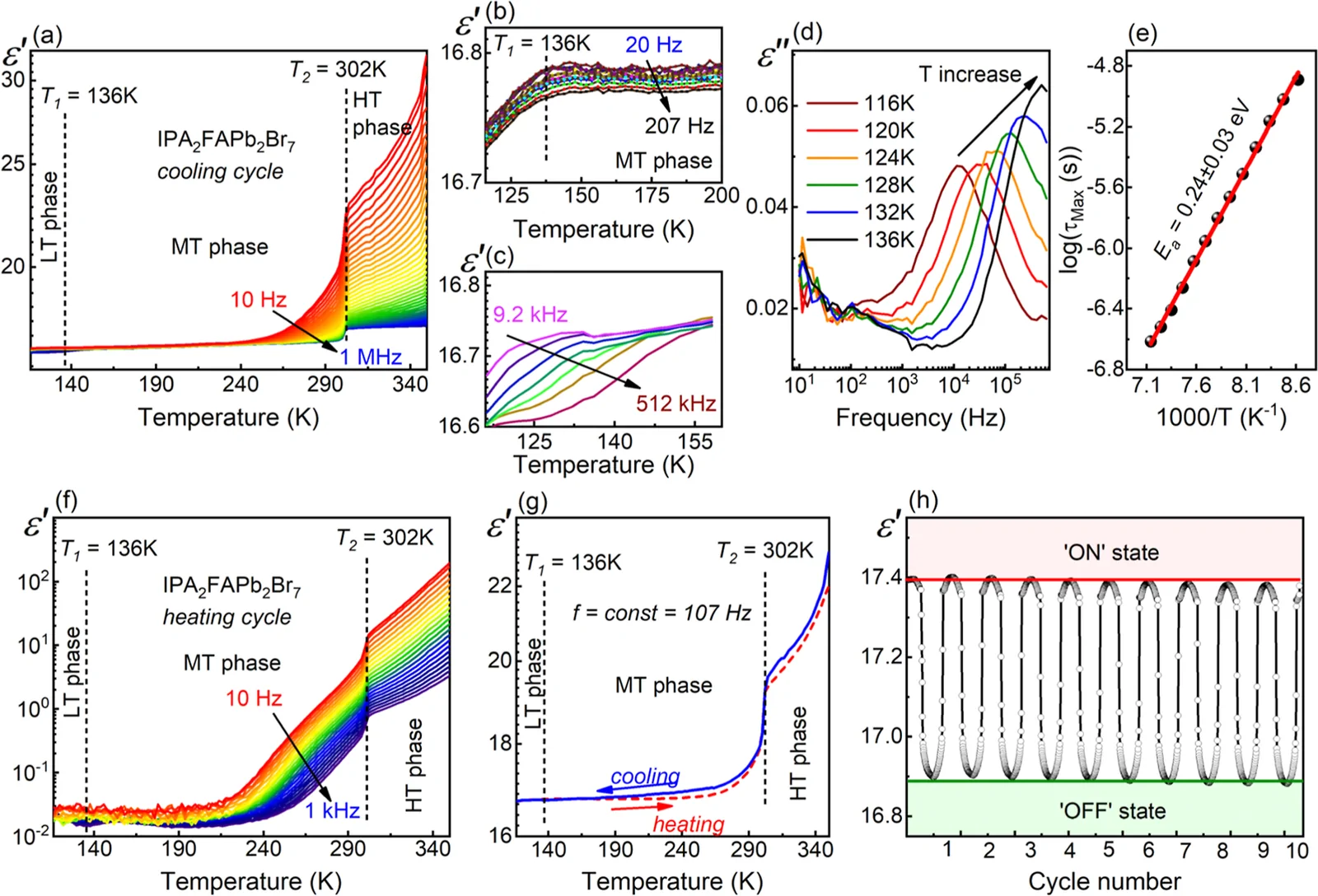
(a) Temperature dependence of ε′ measured between 10 Hz and 1 MHz during a cooling cycle. (b) Dielectric signature of the PT between phases **II** and **III**. (c) Low-temperature dielectric relaxation manifested in ε’. (d) Temperature-induced changes in the relaxation peak visible in the dielectric loss spectra. (e) Temperature dependence of relaxation times parametrized by the Arrhenius law. (f) Imaginary part of the complex dielectric permittivity ε’’ measured between 10 Hz and 1 kHz during a heating cycle. (g) Reversibility of temperature-related changes in ε’. (h) Variation of ε′ measured at 1 MHz as a function of temperature during ten heating–cooling cycles performed between 320 and 280 K.

Further cooling within the phase **II** regime leads to a gradual decrease in ε′, particularly pronounced at lower frequencies down to approximately 250 K. Below this temperature, ε′ stabilizes at about 16.7–16.9 for all probing frequencies. Notably, the structural transformation to the LT phase **III** at 136 K results in a further decrease in ε′. However, this effect is less pronounced compared with that observed at 302 K, and it is most clearly visible in the low-frequency range (20–207 Hz), as illustrated in Figure 6b. In contrast, the high-frequency region is characterized by an additional step-like anomaly that systematically shifts toward lower temperatures with decreasing probing frequency (Figure 6c). This behavior can be attributed to a dielectric relaxation associated with dynamic disorder in the crystal. The relaxation process is even more clearly observed in the frequency-dependent dielectric loss spectra ε″(*f*), where it appears as a single bell-shaped peak that gradually shifts toward lower frequencies upon cooling (Figure 6d). To elucidate the physical origin of this process, the ε″(*f*) spectra were fitted using the Havriliak–Negami model, allowing extraction of the relaxation times as a function of temperature. The fitting was performed using the expression

1 $$\varepsilon^{*}\left(\omega \right)=\varepsilon_{\infty }+\frac{\Delta \varepsilon }{{\left(1+{\left(i\omega \tau_{HN}\right)}^{\alpha_{HN}}\right)}^{\beta_{HN}}}$$

where ε* represents the complex dielectric permittivity, ε_∞_ denotes its high-frequency limit, Δε is the dielectric increment, ω is the angular frequency, and τ_HN_ denotes the so-called Havriliak–Negami characteristic relaxation time. The parameters α_HN_ and β_HN_ characterize the symmetric and asymmetric broadening of the relaxation process, respectively. The relaxation time τ_max_ at a given temperature was subsequently determined based on the relation

2 $$\tau_{\max}=\tau_{HN}{\left[\sin\left(\frac{\alpha_{HN}\pi }{2\beta_{HN}+2}\right)\right]}^{-1/\alpha_{HN}}{\left[\sin\left(\frac{\alpha_{HN}\beta_{HN}\pi }{2\beta_{HN}+2}\right)\right]}^{1/\alpha_{HN}}$$

The determined values of τ_max_ as a function of 1000/T show linear dependence in the LT phase **III** (Figure 6e). Accordingly, the data were fitted using the Arrhenius equation

3 $$\tau_{\max}\left(T\right)=\tau_{0}\exp\left(\frac{E_{a}}{k_{B}T}\right)$$

where τ_0_ denotes the relaxation time at the limit of an infinitely high temperature, *k*_B_ is the Boltzmann constant, and *E*_a_ is the activation energy. The experimental τ_max_(*T*) dependence is accurately parametrized when logτ_0_ and *E*_a_ are approximately −15.3 ± 0.3 and 0.24 ± 0.03 eV, respectively. Such an activation energy is typical for hybrid compounds containing dynamically disordered single-protonated organic cations.^49^ According to our X-ray diffraction and Raman data, only intralayer FA^+^ cations exhibit significant dynamic disorder in phase **II** that strongly slows down in phase **III** and freezes below 80 K. The appearance of dielectric relaxation below the LT PT indicates that these cations remain capable of reorientation in response to the applied electric field even in the LT phase, which is characteristic of persistent orientational disorder.^50^ A similar phenomenon has previously been reported for the one-dimensional hybrid aminoguanidinium lead iodide (AGAPbI_3_).^50^ Notably, the reorientational dynamics in the present compound are significantly faster than in AGAPbI_3._ According to eq 3, the dynamics of FA^+^ cations enter a time scale of ∼100 s (typical for the crossover from dynamic to static disorder^50^) at roughly 70 K. This estimate should, however, be treated with caution since it is based on an extrapolation spanning several decades of relaxation times and assumes that Arrhenius behavior persists below the low limit of the experimental temperature window (116 K). Nevertheless, the presence of dielectric relaxation in the LT phase (below 136 K), its continuous slowing down upon cooling, and the absence of any additional PT below 136 K (as confirmed by our adiabatic measurements) strongly supports the conclusion that the dynamic-to-static crossover may occur within the LT phase. The extrapolated crossover temperature is also consistent, within uncertainty, with the temperature ∼80 K at which narrowing of FA-related Raman bands is observed. The remaining difference between both estimates likely reflects the uncertainty inherent in long-range extrapolation of relaxation times.

Notably, the crossover temperature estimated from the Arrhenius fit is about 100 K lower than that reported for AGAPbI_3_ using the same methodology (∼170–180 K). As a result, in the present compound the relaxation dynamics remain extremely fast even at 130 K, with relaxation times reaching only 1.5 μs. Consequently, it becomes too fast to be detected below 1 MHz when the structure transforms into the intermediate phase **II**.

Instead, in the intermediate phase **II**, as well as in the HT phase **I**, the dielectric loss spectra are dominated by the conductivity contribution, which is also clearly visible in the ε″(*T*) dependence (Figure 6f). In this representation, the PT at 136 K is essentially not detectable, whereas the PT at 302 K appears as a small change in ε″. According to the IR and Raman studies, this effect (together with the frequency-independent step-like change in ε′ at 302 K) can be related to the activation of an additional degree of freedom of the organic ions. Such a shift typically allows for effective tuning of the optoelectronic parameters in hybrid compounds. In IPA_2_FAPb_2_Br_7_, this can be realized by the switching of the dielectric permittivity, as the dielectric permittivity experiences comparable temperature dependences on both cooling and heating cycles (Figure 6g).

Figure 6h shows the variation of ε′ measured at 1 MHz as a function of temperature during the heating–cooling cycles performed between 320 and 280 K. As can be seen, each cooling or heating cycle is accompanied by a sudden and reproducible change in ε′, even after 10 switching events. The dielectric “OFF” state, characterized by ε′ ≈ 16.9, is reached at 280 K, whereas the dielectric “ON” state, with ε′ ≈ 17.4, is obtained at 320 K. Notably, these ε′ values remain stable throughout all the performed switching cycles. However, the dielectric switching mechanism in IPA_2_FAPb_2_Br_7_ differs from that in conventional hybrid perovskites as it involves only partial structural ordering of polar organic cations.

### Optical Properties

One of the hallmark features of hybrid perovskites is bandgap engineering. The RT diffuse reflectance spectrum of IPA_2_FAPb_2_Br_7_ exhibits a pronounced band centered at 439 nm (2.82 eV), which can be attributed to excitonic absorption (Figure S12a). The optical bandgap (*E*_g_) was estimated using the Kubelka–Munk function combined with the Tauc formalism,^51,52^ yielding a value of 2.89 eV (Figure S12b). This bandgap is comparable to the bandgap of IPA_2_TzPb_2_Br_7_ (Tz = 1,2,4-triazolium, *E*_g_ = 2.90 eV),^37^ despite the significantly smaller size of FA^+^ than Tz^+^. Literature data show that the bandgap narrows with decreasing Pb-X bond length and increasing Pb–Br–Pb bond angle.^8,53^ Very similar bandgaps of IPA_2_FAPb_2_Br_7_ and IPA_2_TzPb_2_Br_7_ can be attributed to the fact that the effect of shortening of the Pb–Br bonds due to the replacement of larger Tz^+^ by smaller FA^+^ is compensated by increased Pb–Br–Pb angles from 175.8° to 176.54° for IPA_2_TzPb_2_Br_7_ ^37^ to 153.6–177.0° for IPA_2_FAPb_2_Br_7_.

To obtain information on the temperature dependence of the bandgap, temperature-dependent optical transmittance spectra were measured in the 10–300 K range (Figure 7a). The optical bandgap was determined by linear extrapolation of the steep absorption edge, and the bandgap values were determined as the root of the fitted linear function. The *x*-axis values were calculated from the energy values. Due to the large thickness of the sample (∼200 μm), the *E*_g_ values determined in this way were underestimated. Therefore, the bandgap was constrained by the RT bandgap value obtained from diffuse reflectance measurements, and the obtained offset was applied to the full data set of calculated bandgap values. The temperature dependence of E_g_ is presented in Figure 7b. This figure shows that the bandgap increases with increasing temperature, reaching a maximum value of 2.941 eV at 100 K and significantly decreases on further heating to 2.89 eV at RT. The blue shift in the 10–100 K range suggests that the temperature-induced Pb–Br bond length increase prevails over the decrease of the octahedral distortion (Pb–Br–Pb angle increase), whereas the opposite situation is observed above 100 K. The transmission data show no sudden changes near 130–140 K, which could be attributed to the PT discovered at 136 K. This behavior indicates that the PT weakly affects tilts and distortion of the PbBr_6_ octahedra, in agreement with Raman and X-ray diffraction data.

**Figure 7:**
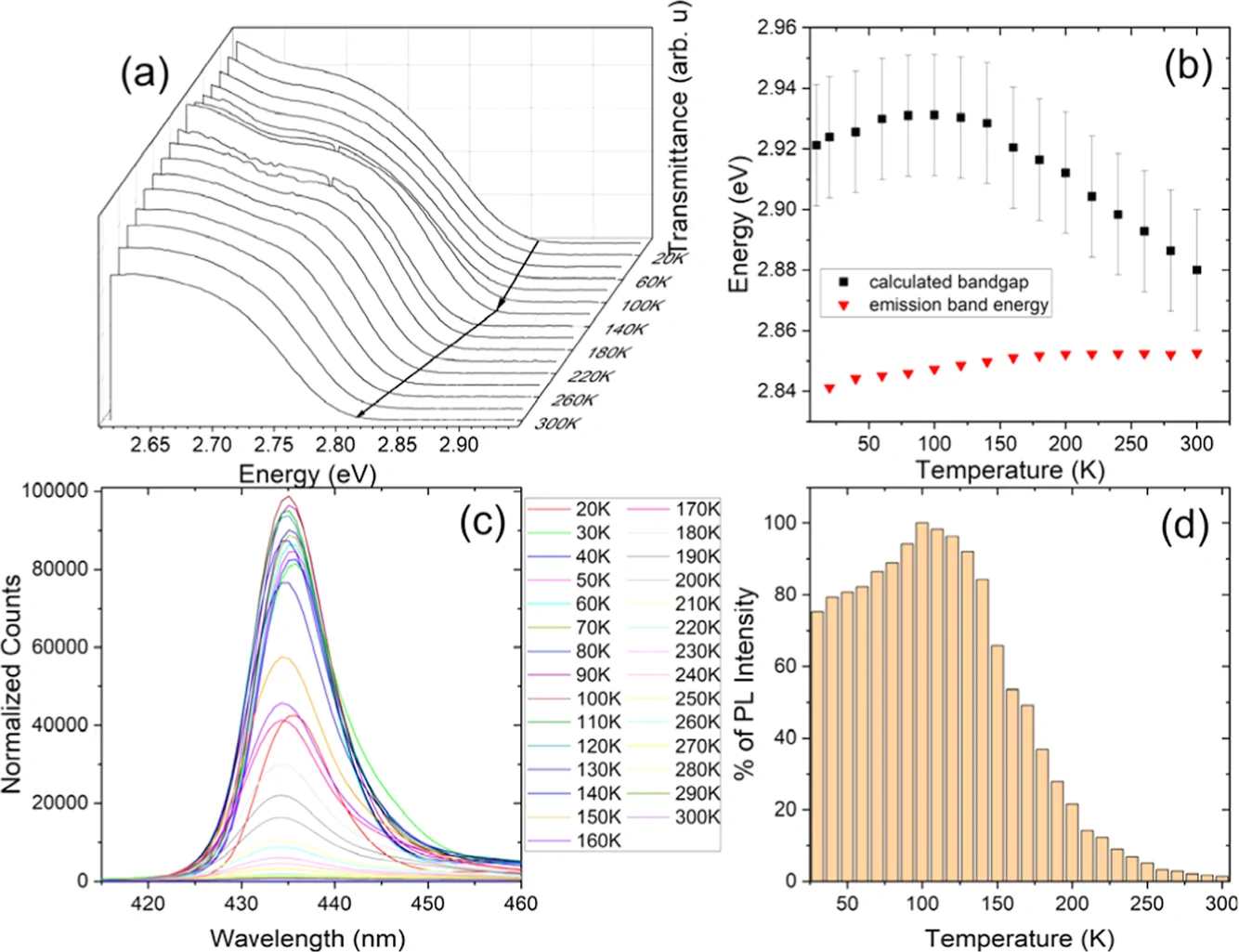
Temperature dependence of (a) transmittance spectra, (b) bandgap and emission band energy, (c) PL spectra, and (d) PL intensity.

The PL spectra exhibit a strong and narrow emission band centered at approximately 435 nm (Figure 7C). There are also several weak, red-shifted bands at approximately 459, 472, 493, 505, 530, and 545 nm (Figure S13). Former studies of mixed-cation perovskites comprising FA^+^ reported that PL of such systems consists of a single band near 526–527 nm, which is strongly red-shifted compared to the excitonic absorption peak.^20,21^ However, it is worth noting that a similar PL band near 528 nm is also reported for nanocrystalline FAPbBr_3_.^54^ Therefore, one of the possible explanations is that the bands near 530 and 545 nm appear due to FAPbBr_3_ impurity, but further studies are needed to unambiguously establish their origin. The remaining weak bands are most likely related to surface defects.

Narrowband emission in hybrid lead halides has usually been described as FE recombination due to its small bandwidth and Stokes shift.^48,53–57^ The FE emission may exhibit both blue or red shifts with increasing temperature, but it should correlate with the behavior of the bandgap and exhibit significant broadening on heating.^48,53–57^ For instance, FE emission of 3D MAPbBr_3_, *n* = 1 2D Tz_2_PbBr_4_, and *n* = 3 (BDA)­(EA)_2_Pb_3_Br_10_ showed blue shift by ∼20, ∼7, and ∼7 nm, respectively, while *n* = 1 MHy_2_PbBr_4_ and *n* = 3 (BDA)­(MA)_2_Pb_3_Br_10_ exhibited red shift by ∼5 nm.^48,53–57^ The red shift of MHy_2_PbBr_4_ PL correlated with the red shift of the bandgap, estimated from the transmission spectra.^51^ As can be seen, the temperature dependence of the PL peak of IPA_2_FAPb_2_Br_7_ does not correlate with the behavior of the bandgap since this emission shows a continuous blue and very weak shift by 1.8 nm (11.4 meV), and the Stokes shift significantly varies as a function of temperature from 94 to 37 meV. Furthermore, full width at half-maximum (fwhm) values exhibit a weak increase on heating from about 8 nm at 20 K to 16.4 nm at 300 K, i.e., fwhm doubles in this temperature range (Figure S14), which is smaller than observed, for instance, in *n* = 2 (PEA)_2_(MA)­Pb_2_I_7_, for which the fwhm value of FE emission increased from 2.7 to 17 nm when going from 80 to 300 K, i.e., fwhm increases more than six times.^58^ It should also be added that the fwhm of IPA_2_FAPb_2_Br_7_ emission bands is much smaller than expected for STE-related emission, for which fwhm usually exceeds 100 nm.^7,10^ Thus, the larger fwhm of the PL band of IPA_2_FAPb_2_Br_7_ at very low temperatures and the weaker temperature dependence of fwhm compared to FE-related PL are consistent with the tentative assignment of IPA_2_FAPb_2_Br_7_ emission to radiative recombination from localized states induced by bandgap fluctuations. Those fluctuations within the crystal might be possible, for instance, due to point defects formed in the crystals by the location of some FA^+^ cations in the interlayer space. Such defects are likely since former studies showed that FA^+^ can also be located as space cations, as evidenced by the formation of 2D FA_2_PbBr_4_.^59^ The effect of such point defects might be similar to the effect of vacancies or dislocations within the inorganic semiconductor structures, inducing change in the bandgap significant enough to allow for localization of excitons.

The temperature-dependent integrated PL intensity shows an initial increase from 20 to 100 K, followed by a gradual decrease at higher temperatures, which indicates the onset of thermal quenching (Figure 7d). The initial increase suggests a temperature-dependent redistribution of the excited-state population or thermally assisted population of the emissive state. Because of this, the low-temperature behavior cannot be described by a simple thermal-quenching model, and only the HT region above the intensity maximum was used to determine the activation energy (*E*_a_).

The maximum PL intensity observed at 100 K was taken as the reference intensity I_0_ for the analysis of the subsequent high-temperature quenching regime. The *E*_a_ was determined using an Arrhenius-type approach based on the linear dependence between the logarithmic intensity ratio and the inverse temperature (1/*T*) (Figure S15). The resulting value of 142 meV should therefore be regarded as an effective activation energy associated with the process governing high-temperature PL quenching rather than as a direct measure of the exciton binding energy, exciton localization energy, or energetic depth of a specific defect state.

If observed emission comes from the mentioned theory of localized emissive states, the extracted activation energy may reflect the thermally assisted escape, delocalization of excitons, or charge carriers from these states. Alternatively, or simultaneously, it may correspond to the activation of competing nonradiative recombination pathways similar to those observed for FE emission. Importantly, the value of 142 meV describes only the dominant quenching process above 100 K and does not account for the initial increase in the PL intensity between 20 and 100 K. The latter may result from thermally assisted carrier transfer from weakly emissive or nonemissive states, carrier detrapping followed by capture at emissive sites, or enhanced migration toward local potential minima.

What supports the mentioned explanation is considerably higher activation energy than is reported for the temperature-dependent quenching of narrow-band emission in *n* = 3 (BDA)­(EA)_2_Pb_3_Br_10_ and (BDA)­(MA)_2_Pb_3_Br_10_ perovskites that yielded values of 41 and 53, respectively.^48^ This difference indicates that PL quenching in the present compound may involve a different thermally activated mechanism than that described for FEs. Nevertheless, the microscopic origin of the extracted activation energy cannot be established unambiguously from steady-state PL measurements alone. Temperature-dependent time-resolved PL measurements would be particularly valuable for distinguishing among FE, localized-state, and defect-related recombination pathways and for clarifying the physical origin of both the low-temperature intensity increase and the high-temperature PL quenching process.

## Conclusions

In summary, a new multifunctional RP bilayered hybrid perovskite, IPA_2_FAPb_2_Br_7_, was successfully synthesized. This perovskite undergoes two structural PTs associated with lowering of crystal symmetry, pronounced tilting of PbBr_6_ octahedra, and only partial ordering of organic cations at the higher-temperature PT, followed by final ordering of FA^+^ cations in the LT phase **III**. Crystallographic data also show that the interlayer IPA^+^ cations exhibit static positional disorder in phases **II** and **III**. Such ordering behavior, different from typical hybrid perovskites, leads to near-room-temperature dielectric switching with a moderate permittivity contrast and low dielectric losses below 225 K. It also results in persistent structural disorder, allowing IPA_2_FAPb_2_Br_7_ to relax even in the LT **III** and enabling continuous modulation of its optoelectronic properties. In this context, the compound exhibits semiconductor properties with a bandgap near 2.9 eV and narrowband blue PL. We also show that the widely used assumption that the temperature dependence of narrow PL reflects the behavior of the bandgap in hybrid lead halides may not be valid in many cases. This is evidenced by the different temperature dependence of the bandgap established from temperature-dependent transmission studies and the position of the narrow PL peak.

Overall, this work reports novel multifunctional perovskites and highlights the importance of temperature-dependent IR and optical transmission studies for a proper understanding of lattice dynamics, PT mechanisms, and optoelectronic properties of hybrid perovskites.

## Supplementary Material



## Data Availability

Data for this article, including BDS data (txt.); DSC data (txt); PL data (dat.); Raman and IR data (opju.) are available at 10.5281/zenodo.19664147.

## Notes

Additional experimental details, including crystallographic data, Raman and IR wavenumbers, X-ray powder diffraction patterns, DSC traces, Raman, IR and diffuse absorption spectra, and Tauc plots and activation energy for thermal quenching (PDF)
