# Supplementary material for: Bilayered Two-Dimensional Hybrid Perovskite with Switchable Dielectric Properties, Persistent Disorder, and Narrow-Band Emission
Source: Inorg Chem. 2026 Aug 4;65(32):18807–18. doi: 10.1021/acs.inorgchem.6c02525 (PMC13488398; doi:10.1021/acs.inorgchem.6c02525)
Supplement: Supplementary file 1 [file ic6c02525_si_001.pdf]

Supporting Information for

Bilayered Two-Dimensional Hybrid Perovskite with Switchable Dielectric Properties, Persistent Disorder and Narrow-Band Emission

Jan Kudrawiec,[a*] Mirosław Mączka,[a] Andrzej Nowok,[b] Anna Gągor,[a] Rafał Bartoszewicz,[c] Daria Szewczyk,[a] Adam Sieradzki[b]

[a]*Institute of Low Temperature and Structure Research, Polish Academy of Sciences, ul. Okólna 2, 50-422 Wrocław, Poland*

[b]*Department of Experimental Physics, Wrocław University of Science and Technology, Wybrzeże Wyspiańskiego 27, 50-370 Wrocław, Poland*

[c]*Department of Semiconductor Materials Engineering, Faculty of Fundamental Problems of Technology, Wrocław University of Science and Technology, Wybrzeże Wyspiańskiego 27, 50-370 Wrocław, Poland*

*Email: j.kudrawiec@intibs.pl

Table S1. X-ray diffraction experimental details. For all structures: M_r = 1139.06. Experiments were carried out with Mo *K*α radiation using a Xcalibur, Atlas. Absorption correction: Multi-scan *CrysAlis PRO* 1.171.38.43 (Rigaku Oxford Diffraction, 2015) Empirical absorption correction using spherical harmonics, implemented in SCALE3 ABSPACK scaling algorithm.

	**Phase I**	**Phase II**	**Phase III**
Crystal data			
Chemical formula		$Br_7Pb_2 \cdot 2(C_3H_{10}N) \cdot CH_5N_2$	
Crystal system, space group	Tetragonal, *I*4/*mmm*	Monoclinic, $P2_1/c$	Monoclinic, $P2_1/c$
Temperature (K)	330	200	95
a, *b*, *c* (Å)	6.0173 (4), 6.0173 (4), 34.761 (6)	17.246 (2), 8.5597 (9), 16.9630 (17)	17.095 (2), 8.4820 (9), 16.9306 (17)
β, (°)	90, 90, 90	90, 103.816 (10), 90	90, 104.219 (10), 90
V (Å^3)	1258.6 (3)	2431.7 (5)	2379.7 (5)
Z	2	4	4
μ (mm^{-1})	24.47	25.33	25.88
Crystal size (mm)	0.24 × 0.14 × 0.03	0.24 × 0.14 × 0.03	0.24 × 0.14 × 0.03
Data collection			
No. of measured, ind. and observed [$I > 2\sigma(I)$] refl.	7393, 569, 432	7269, 7269, 3576	7554, 7554, 3056
R_{int}	0.061	--	--
$(\sin \theta/\lambda)_{max}$ (Å^{-1})	0.688	0.610	0.610
Refinement			
$R[F^2 > 2\sigma(F^2)]$, $wR(F^2)$, S	0.047, 0.134, 1.09	0.057, 0.156, 0.90	0.055, 0.138, 0.84
No. of reflections	569	7269	7554
No. of parameters	33	167	179
No. of restraints	2	28	38
H-atom treatment	–	H-atom parameters constrained	H-atom parameters constrained
$\Delta\rho_{max}$, $\Delta\rho_{min}$ (e Å^{-3})	1.54, -0.97	3.01, -2.14	3.47, -3.45

Computer programs: *CrysAlis PRO* 1.171.42.93a (Rigaku OD, 2023), *SHELXT* (Sheldrick, 2015), *SHELXL* 2018/3 (Sheldrick, 2015), Olex2 1.5 (Dolomanov *et al.*, 2009).

The crystal structures were determined from very small and thin single-crystal domains, which were necessary to isolate single-domain regions. Consequently, the diffraction intensities were relatively weak, resulting in lower redundancy and lower observed/unique reflection ratios. In addition, both low-temperature phases were refined as two-component twins. Twinning caused partial overlap and splitting of

Bragg reflections, further reducing the effective intensity and data quality.

The IPA cation exhibits positional disorder in the low-temperature phases, where split carbon sites with partial occupancies reduce the precision of C–C bond lengths. In the high-temperature phase, the disorder is even more pronounced, with multiple split positions of both molecular cations. As a consequence, hydrogen atoms were not generated for the highly disordered organic fragments, which explains the formula and molecular-weight discrepancies reported by checkCIF.

These limitations primarily affect the precision of the geometry of the disordered organic cation. However, they do not influence the determination of the inorganic Pb–Br framework, the crystal symmetry, phase assignment, or the structural changes discussed in the manuscript. The conclusions regarding the phase transitions and the evolution of the layered perovskite framework are therefore robust despite the crystallographic limitations associated with disorder, twinning, and weak diffraction.

Table S2. Pb-Br distances in $IPA_2FAPb_2Br_7$ (Å).

Phase I			
Pb1—Br2^{i}	3.0090 (2)	Pb1—Br1	2.862 (5)
Pb1—Br2	3.0090 (2)	Pb1—Br1^{ii}	2.862 (5)
Pb1—Br2^{ii}	3.0090 (2)	Pb1—Br1^{iv}	2.862 (5)
Pb1—Br2^{iii}	3.0090 (2)	Pb1—Br1^{v}	2.862 (5)
Pb1—Br3	3.0890 (10)		

Phase II			
Pb1—Br1	3.070 (2)	Pb2—Br1^{viii}	3.122 (2)
Pb1—Br6	3.002 (2)	Pb2—Br6^{ix}	3.034 (2)
Pb1—Br2	2.986 (2)	Pb2—Br2	2.978 (2)
Pb1—Br4^{vi}	3.117 (2)	Pb2—Br4	3.016 (2)
Pb1—Br3^{vii}	3.104 (5)	Pb2—Br3	3.080 (5)
Pb1—Br7	2.839 (3)	Pb2—Br8	2.860 (3)

Phase III			
Pb1—Br1	3.062 (2)	Pb2—Br1^{viii}	3.110 (2)
Pb1—Br6	2.981 (2)	Pb2—Br6^{ix}	3.010 (2)
Pb1—Br2	2.965 (2)	Pb2—Br2	2.972 (2)
Pb1—Br4^{vi}	3.116 (2)	Pb2—Br4	3.000 (2)
Pb1—Br3^{vii}	3.094 (3)	Pb2—Br3	3.075 (3)
Pb1—Br7	2.824 (3)	Pb2—Br8	2.849 (3)

Symmetry code(s): (i) *x*, *y*+1, *z*; (ii) -*y*+1, *x*, *z*; (iii) -*y*, *x*, *z*; (iv) -*x*+1, -*y*+1, *z*; (v) *y*, -*x*+1, *z*; (vi) *x*, *y*-1, *z*; (vii) -*x*+2, -*y*+1, -*z*+1; (viii) *x*, -*y*+1/2, *z*-1/2; (ix) *x*, -*y*+3/2, *z*-1/2.

Table 3. Selected hydrogen-bond parameters in ordered phase III, T=95 K.

D—H···*A*	*D*—H (Å)	H···*A* (Å)	*D*···*A* (Å)	*D*—H···*A* (°)
N12—H12D···Br4^{i}	0.88	2.95	3.57 (2)	129.1
N12—H12D···Br3^{ii}	0.88	2.97	3.600 (18)	130.1
N12—H12E···Br2	0.88	3.02	3.57 (2)	122.7
N12—H12E···Br3	0.88	2.86	3.55 (2)	136.6
N11—H11A···Br4^{ii}	0.88	2.97	3.59 (2)	128.6
N11—H11A···Br3^{i}	0.88	2.96	3.62 (2)	132.6
N11—H11B···Br3^{iii}	0.88	2.72	3.46 (2)	142.0
N1—H1A···Br7^{iv}	0.91	2.56	3.380 (17)	150.1
N1—H1B···Br1^{v}	0.91	2.68	3.580 (17)	169.6
N1—H1C···Br6^{v}	0.91	2.92	3.648 (18)	138.2
N1—H1C···Br8^{vi}	0.91	2.73	3.394 (17)	130.2
N2—H2BD···Br8	0.91	2.73	3.497 (16)	142.2
N2—H2BE···Br6	0.91	2.75	3.636 (17)	165.4
N2—H2AA···Br7^{vii}	0.91	2.79	3.519 (17)	138.0
N2—H2AB···Br2	0.91	2.71	3.618 (16)	177.2
N2—H2AC···Br1^{vii}	0.91	2.66	3.545 (16)	165.7

Symmetry code(s): (i) *x*, *y*-1, *z*; (ii) -*x*+2, -*y*+1, -*z*+1; (iii) -*x*+2, *y*-1/2, -*z*+1/2; (iv) -*x*+1, *y*+1/2, -*z*+1/2; (v) -*x*+1, -*y*+1, -*z*+1; (vi) -*x*+1, *y*-1/2, -*z*+1/2; (vii) *x*, *y*+1, *z*.

Table S4. Raman wavenumbers (in cm^{-1}) of powdered $IPA_2FAPb_2Br_7$ sample measured at RT using FT-Raman spectrometer (λ_{exc}=1064 nm) and of single $IPA_2FAPb_2Br_7$ crystal at 320, 160 and 80 K measured using Raman microscope (λ_{exc}=830 nm) together with the proposed assignment.[a] Modes corresponding to FA^+ are in bold.

RT FT-Raman	320 K	160 K	80 K	assignment
**3083w**				**$\nu_s(NH_2)$**
2984s				$\nu_s(CH_3)$
2940s				$\nu_s(CH_3)$
2915w				$\nu_{as}(CH_3)$
2900w				combination
**1718vw**				**$\nu_{as}(CN)$**
**1617w**				**$\delta(NH_2)$**
1582m	1578w	1581m	1582m	$\delta_{as}(NH_3)$
1570sh				$\delta_{as}(NH_3)$
1462s	1463w	1467w	1468w	$\delta_s(NH_3)$
1446m	1443w			$\delta_{as}(CH_3)$
**1396m**	**1394m**	**1392w**	**1389w**	**$\delta(CH)$**
1374m				$\delta_s(CH_3)$
1369m				$\delta_s(CH_3)$
1351w				$\delta(CH)$
1346w				$\delta(CH)$
1204m	1204m	1213w+1204w	1211w+1205w	$\rho(CH_3)$
1157w				$\rho(CH_3)$
**1114w,b**				**$\rho(NH_2)$**
985sh				$\nu(CN)$
977m	978w	975w		$\nu(CN)$
956w				$\rho(CH_3)$
924m	925m	925w	926w	$\rho(CH_3)$
796s	795m	798m	797m	$\nu_s(CCC)$

**604w**				**τ(NH_2)**
**522s**	**520s**	**520m**	**526m+514s**	**δ(NCN)**
459w	457w	451w	450w	δ(CCN)
393m	392m	387m	388w	γ(CCN)
380w				δ_s(CCC)
		302w	300w	τ(CH_3)
			**280w**	**FA-cage mode**
256sh	254sh	255w,b	254m	τ(CH_3)
		210w	214m	L+ T'+τ(CH_3)
	152sh	160m	161w	L+ T'+ Pb-Br stretch
	119sh	124m	132m+123m	L+ T'+ Pb-Br stretch
	88sh	95m	97m	L+ T'+ Pb-Br stretch
		72m	71m	Pb-Br bend
	48vs	57vs+47vs	58vs+50vs	Pb-Br bend
	31vs	35vs+30w	36vs+30w	L($PbBr_6$)
		23s	24s	L($PbBr_6$)

[a] key: vs, very strong; s, strong; m, medium; w, weak; vw, very weak; sh, shoulder; ν, stretching; δ, in-plane bending; γ, out-of-plane bending; ρ, rocking; τ, twist; L, librational mode; T', translational mode of organic cation.

Table S5. FT-IR wavenumbers (in cm^{-1}) of powdered $IPA_2FAPb_2Br_7$ sample measured at 320, 160 and 7 K together with the proposed assignment.[a] Modes corresponding to FA^+ are in bold.

320 K	160 K	7 K	assignment
**3428m**	**3428m**	**3428m**	**$\nu_{as}(NH_2)$**
**3399s**	**3403s**	**3411m+3391s**	**$\nu_{as}(NH_2)$**
**3356m**	**3350m**	**3352w**	**$\nu_{as}(NH_2)$**
**3328sh**	**3325sh**	**3339w+3331w**	**$\nu_s(NH_2)$**
		**3308w+3300w**	**$\nu_s(NH_2)$**
**3259m**	**3267m**	**3274m+3245w+3233w**	**$\nu_s(NH_2)$**
3172s	3176s+3164s+3146s	3192sh+3167m+3158w+3143s	$\nu_{as}(NH_3)$
**3128s**	**3117s**	**3123sh+3118s**	**$\nu_s(NH_2)$**
**3080sh**	**3082w**	**3081m+3071w**	**$\nu_s(NH_2)$**
3030sh	3032w	3040w_3031w	$\nu_{as}(CH_3)$
2980w	2981w	2983w +2977w	$\nu_s(CH_3)$
2922w	2936w+2921w	2936w +2918w	$\nu_s(CH_3)$
		**1717vs**	**$\nu_{as}(CN)$**
**1685vw**	**1684vw**	**1683vw**	**$\delta(NH_2)$**
		**1642w**	**$\delta(NH_2)$**
**1616m**	**1606m**	**1617w+1597m**	**$\delta(NH_2)$**
1577s	1576s+1561w	1574m+1559m	$\delta_{as}(NH_3)$
1476s	1478m	1474m	$\delta_s(NH_3)$
1464m	1465s+1459m	1464s+1458m	$\delta_{as}(CH_3)$
1398s	1400w+1396m+1392s	1401w+1396m+1391s	$\delta_s(CH_3)$
1381s	1380s	1383w+1380s+1378sh	$\delta_s(CH_3)$
1371w	1372vw+1364w	1372vw+1364w	$\delta_s(CH_3)$
1356w	1353w	1351w	$\delta(CH)$
1346w	1347w+1330vw	1348w+1334w	$\delta(CH)$
	**1303vw,b**	**1301w**	**$\rho(NH_2)$**
		**1242w**	**$\rho(NH_2)$**
1208sh	1212sh	1214m	$\rho(CH_3)$

1202s	1205s	1207s+1205s	ρ(CH_3)
1156m	1164m+1153w	1165s+1153w	ρ(CH_3)
	**1107w,b**	**1110w+1100w**	**ρ(NH_2)**
**1056m**	**1055m**	**1061m+1054m**	**ν(CN)**
**1018w**	**1020w**	**1025w+1018m+1016w+1013m**	**ν(CN)**
984sh	988m	991m+986m	ν(CN)
976m	972s	977sh+973s	ν(CN)
955vw	955w	959sh+956w	ρ(CH_3)
935w,b	934w	934w	ρ(CH_3)
924w	927w+924sh	928sh+926m+923m	ρ(CH_3)
794w	796w	797m+795m	ν_s(CCC)
	**724vw,b**	**725vw**	**ω(NH_2)**
**625s,b**	**628s,b**	**651m+628s+621s+604w**	**τ(NH_2)**
**521vw**	**520vw+515w**	**526vw+522vw+510sh+507w**	**δ(NCN)**
457w	456w+448w	459w+457w+451sh+447w	δ(CCN)

[a] key: vs, very strong; s, strong; m, medium; w, weak; vw, very weak; sh, shoulder; ν, stretching; δ, in-plane bending; γ, out-of-plane bending; ρ, rocking; τ, twist; L, librational mode; T', translational mode of organic cation.

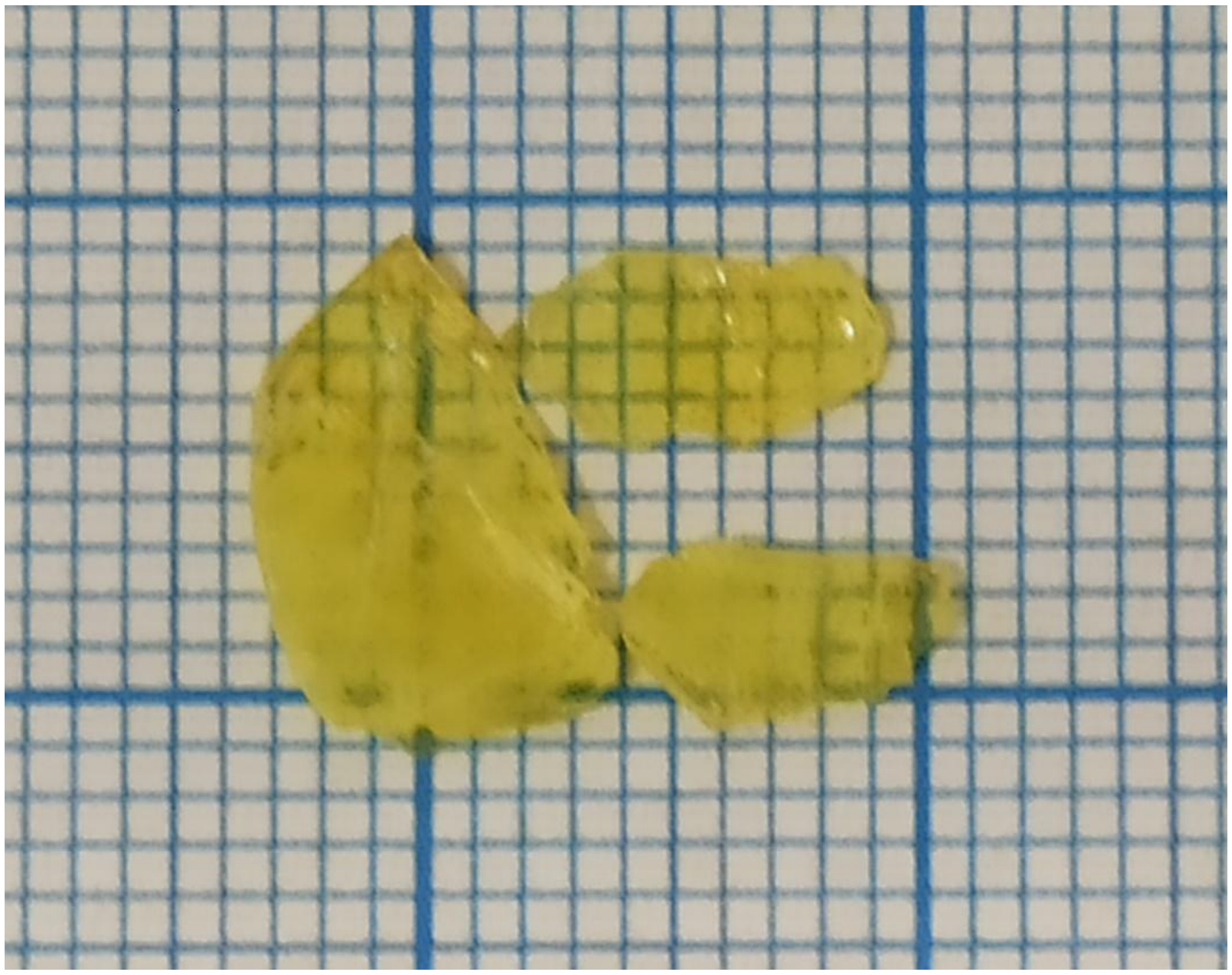

Figure S1. Photos of $IPA_2FAPb_2Br_7$ crystals.

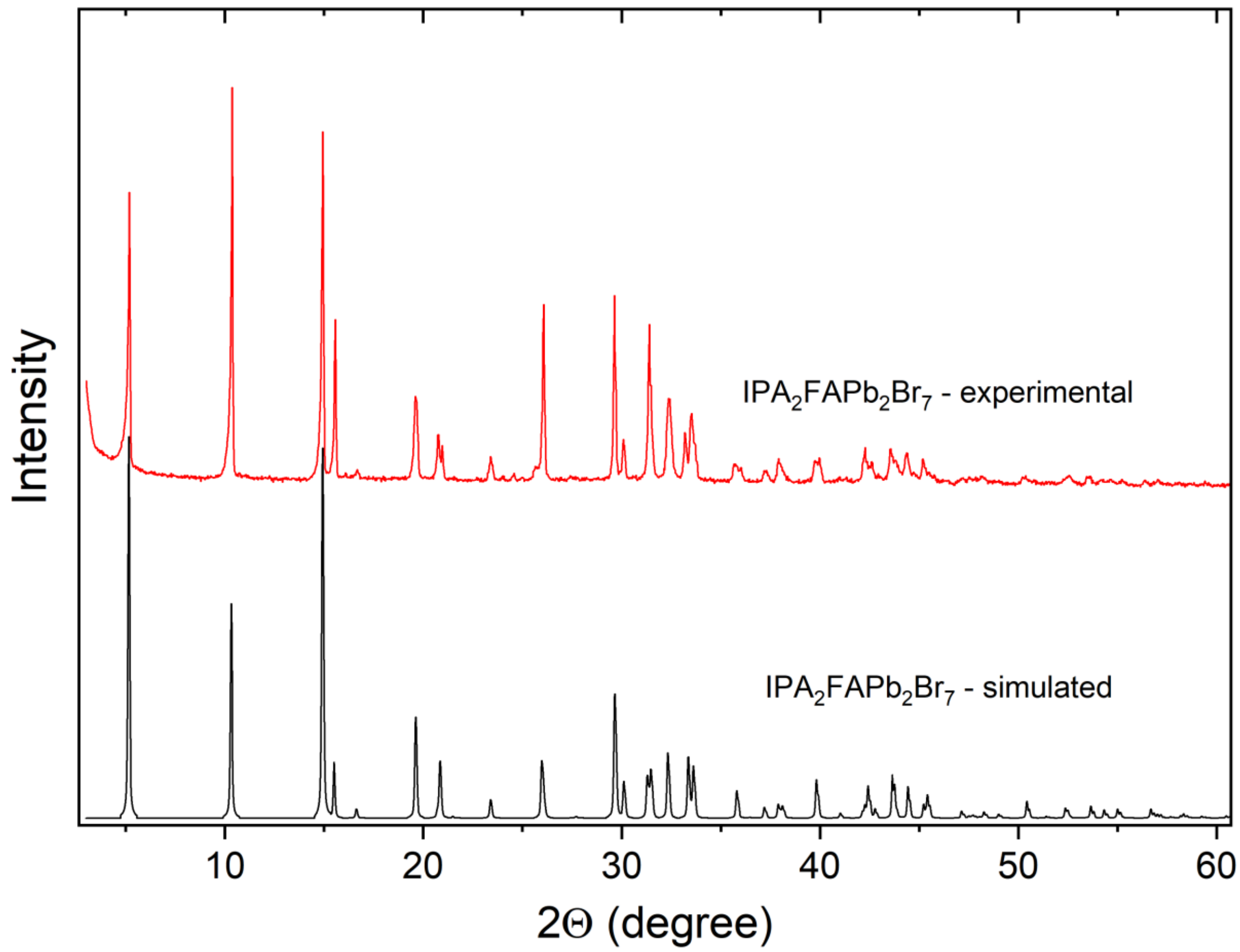

Figure S2. Experimental and simulated powder diffraction patterns of $IPA_2FAPb_2Br_7$.

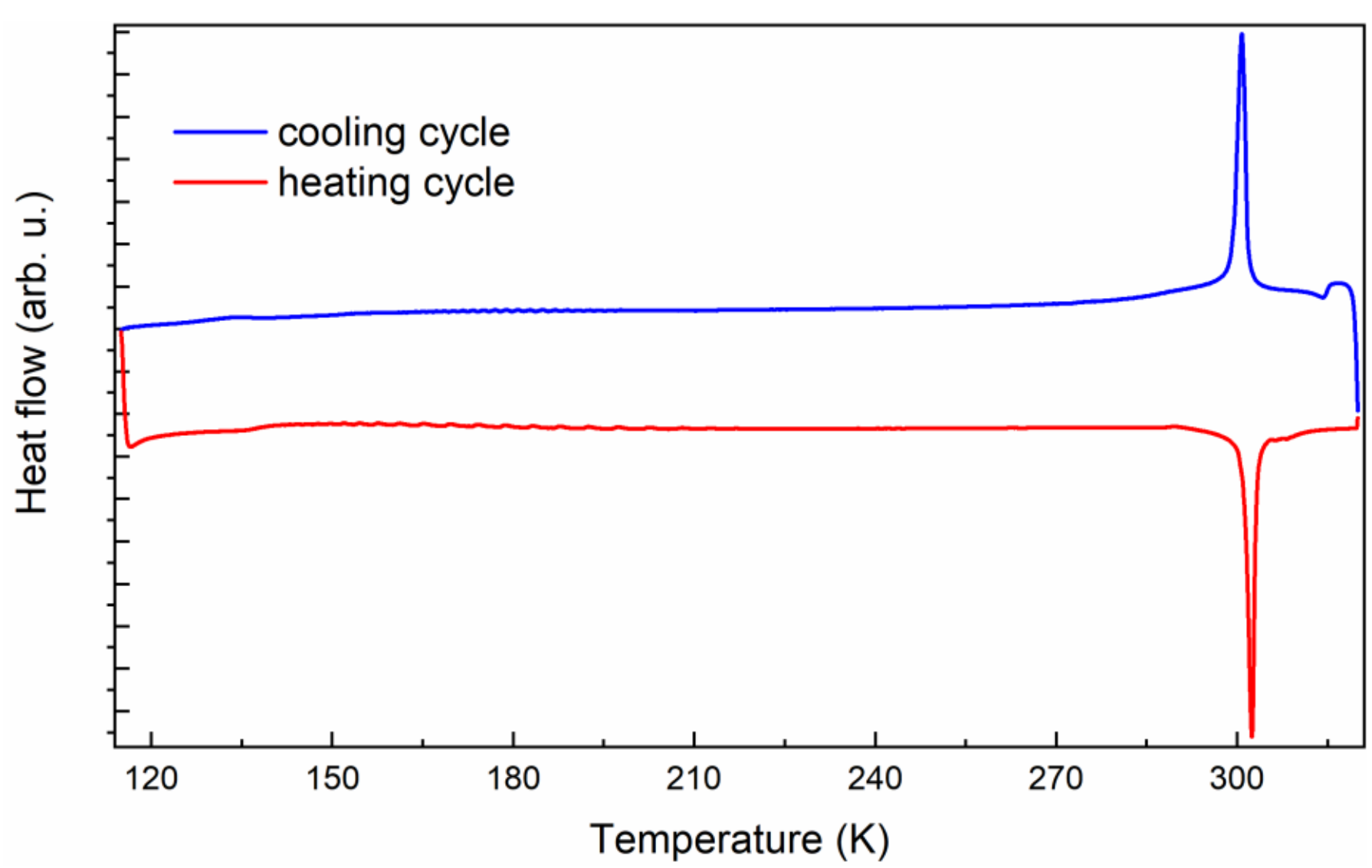

Figure S3. DSC traces for $IPA_2FAPb_2Br_7$.

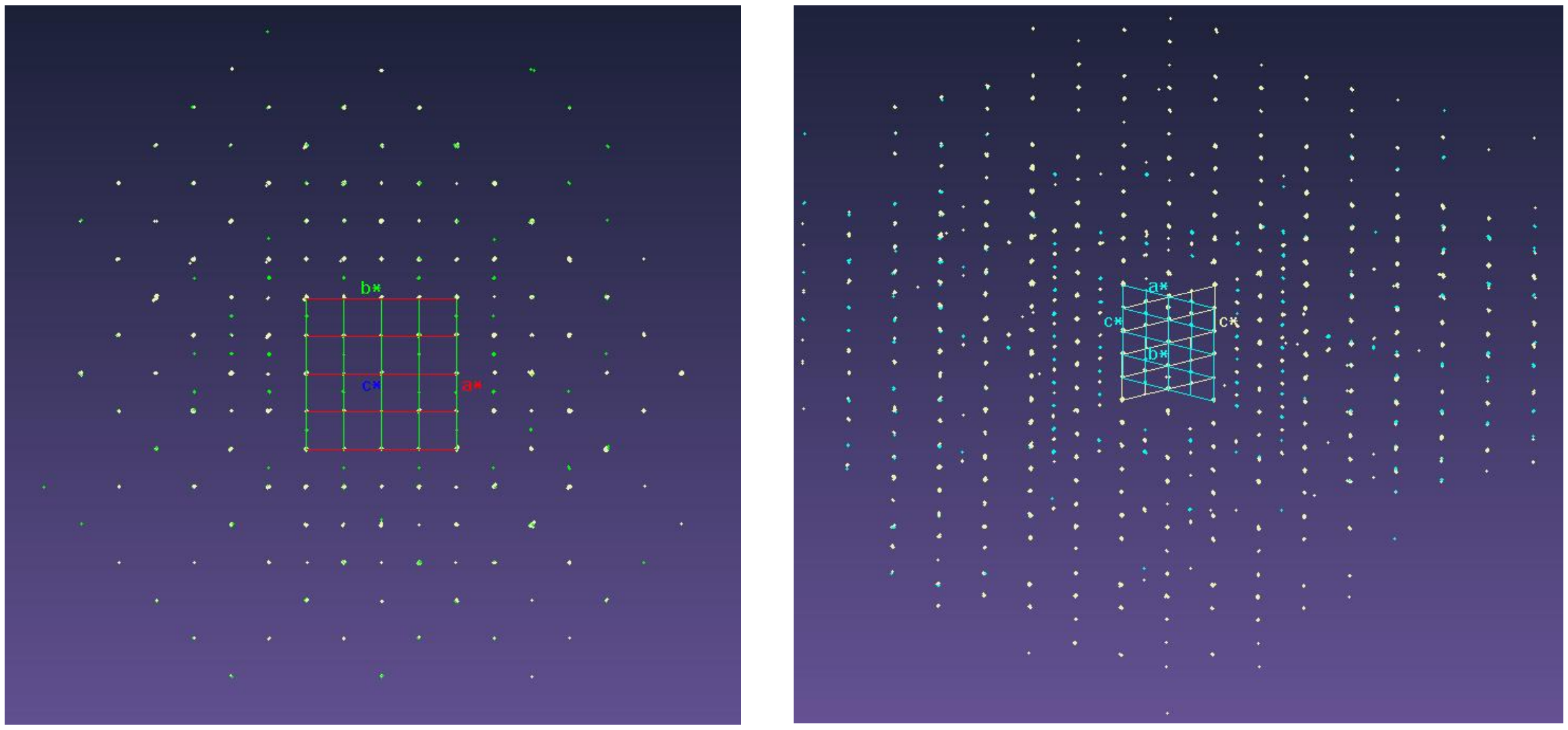

Figure S4. Ewald sphere from x-ray diffraction data collected at RT (left) and 95 K (right), showing pseudo-tetragonal diffraction patterns at RT with low-intensity peaks (green) from twin domains and two, well separated twin-domains with partially overlapped diffraction peaks at 95 K.

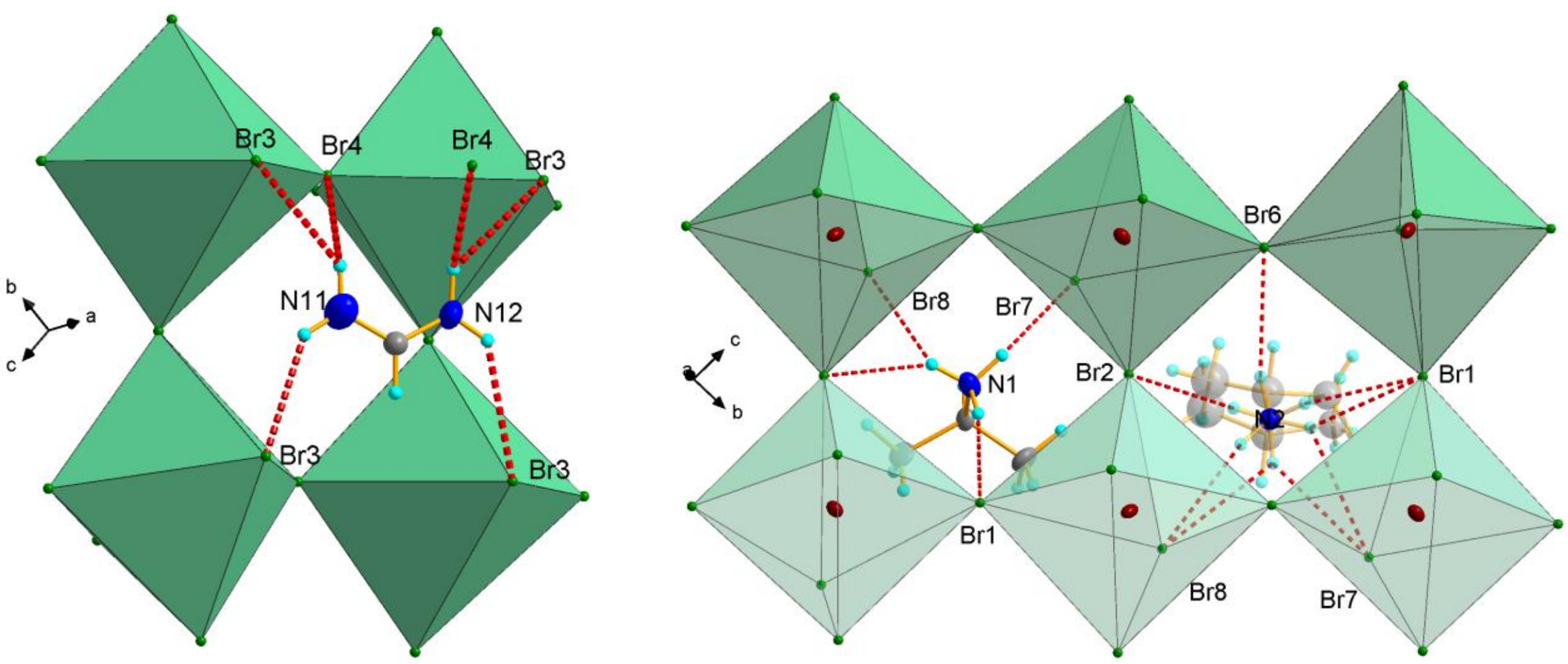

Figure S5. Hydrogen bonds in Phase III, T=95 K, atom numbering for Table S3.

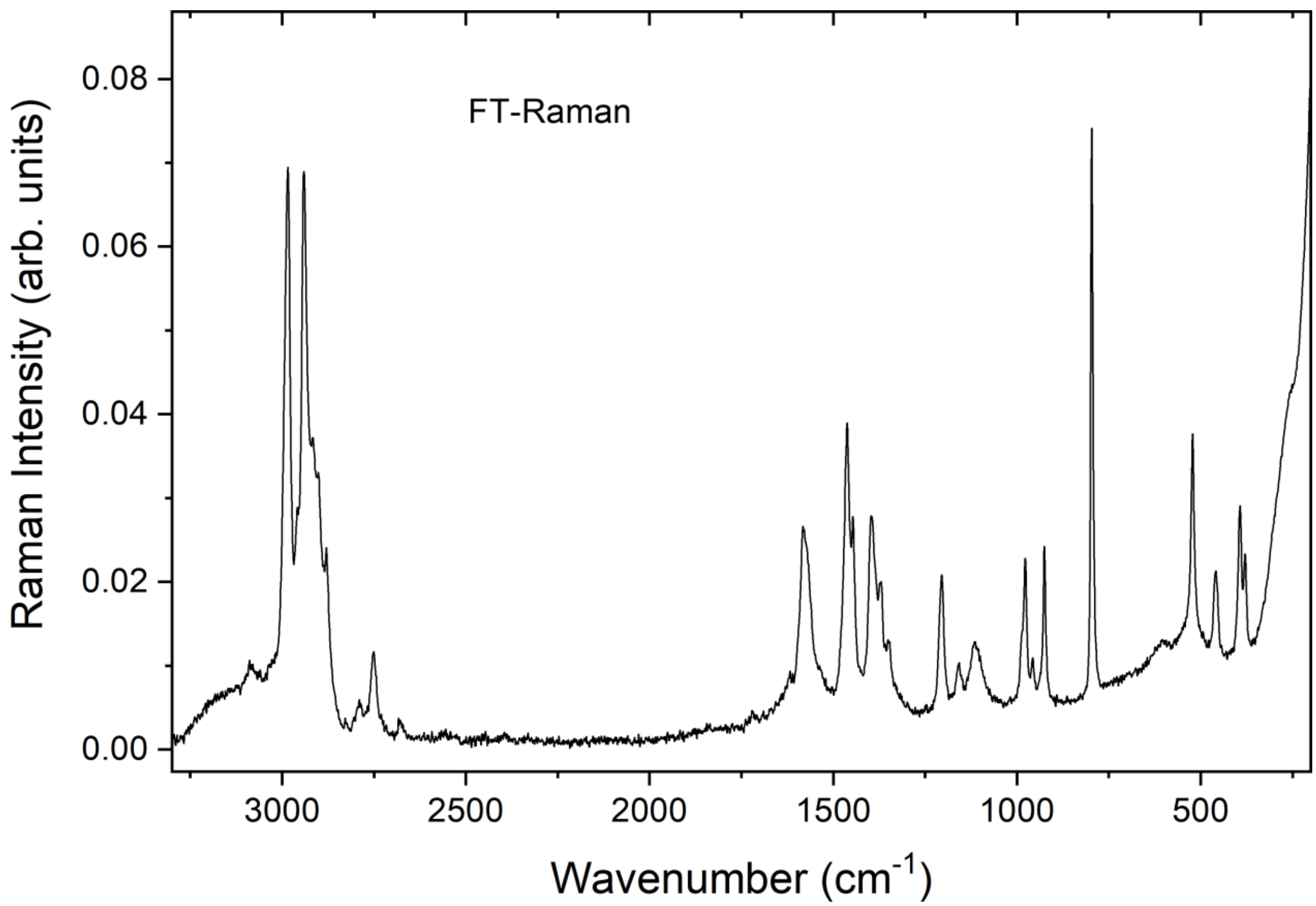

Figure S6. RT Raman spectrum of $IPA_2FAPb_2Br_7$ in the 3300-200 cm^{-1} range measured using FT-Raman spectrometer and 1064 nm excitation.

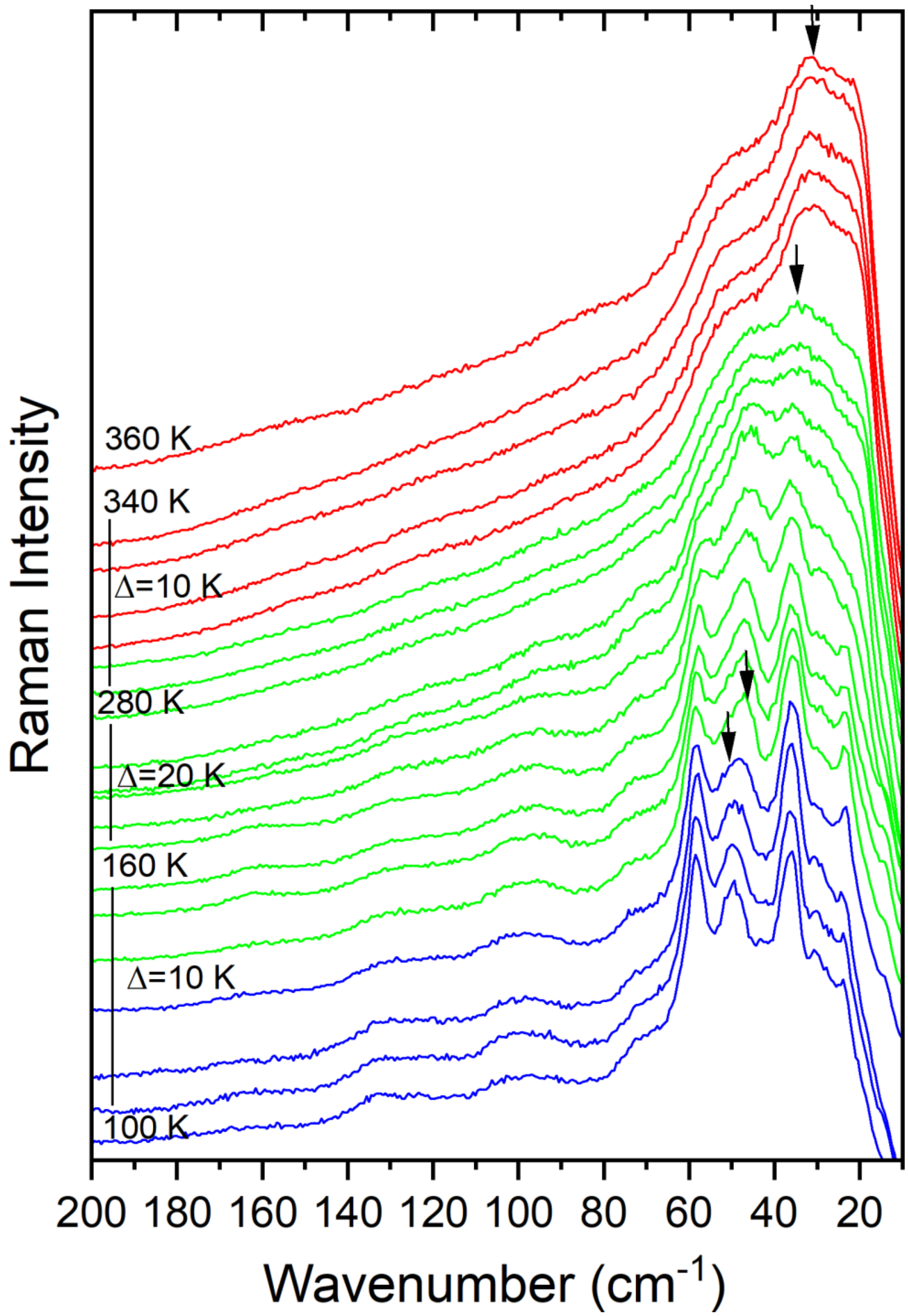

Figure S7. Temperature-dependent Raman spectra of $IPA_2FAPb_2Br_7$ in the (a) 600-58 cm^{-1} and (b) 100-10 cm^{-1} range measured in the Linkam cell. Arrows indicate the Pb-Br bend mode exhibiting significant shifts at the PTs.

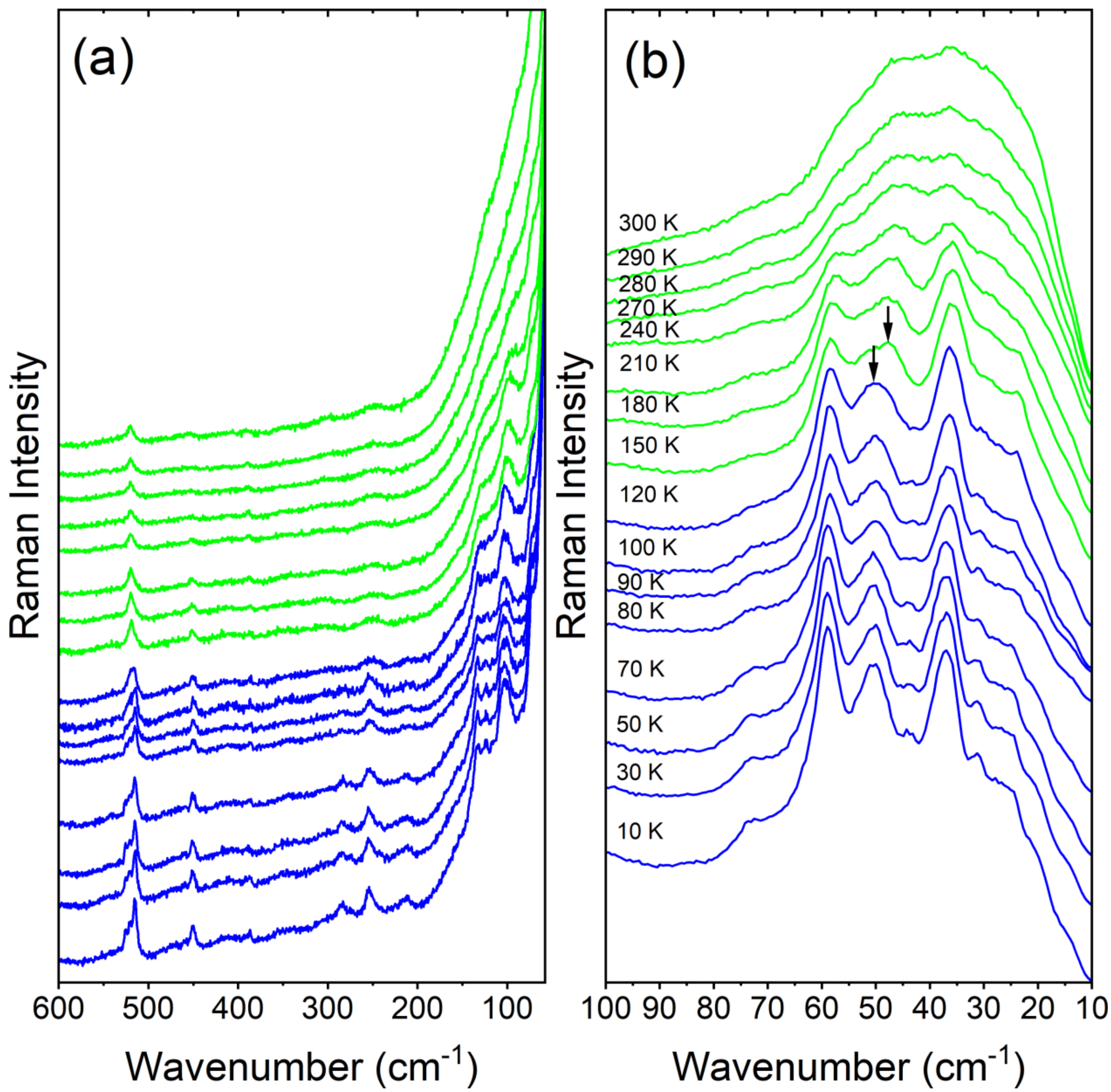

Figure S8. Temperature-dependent Raman spectra of $IPA_2FAPb_2Br_7$ in the (a) 600-58 cm^{-1} and (b) 100-10 cm^{-1} range measured in the cryostat. Arrows indicate the Pb-Br bend mode exhibiting significant shifts at the PTs.

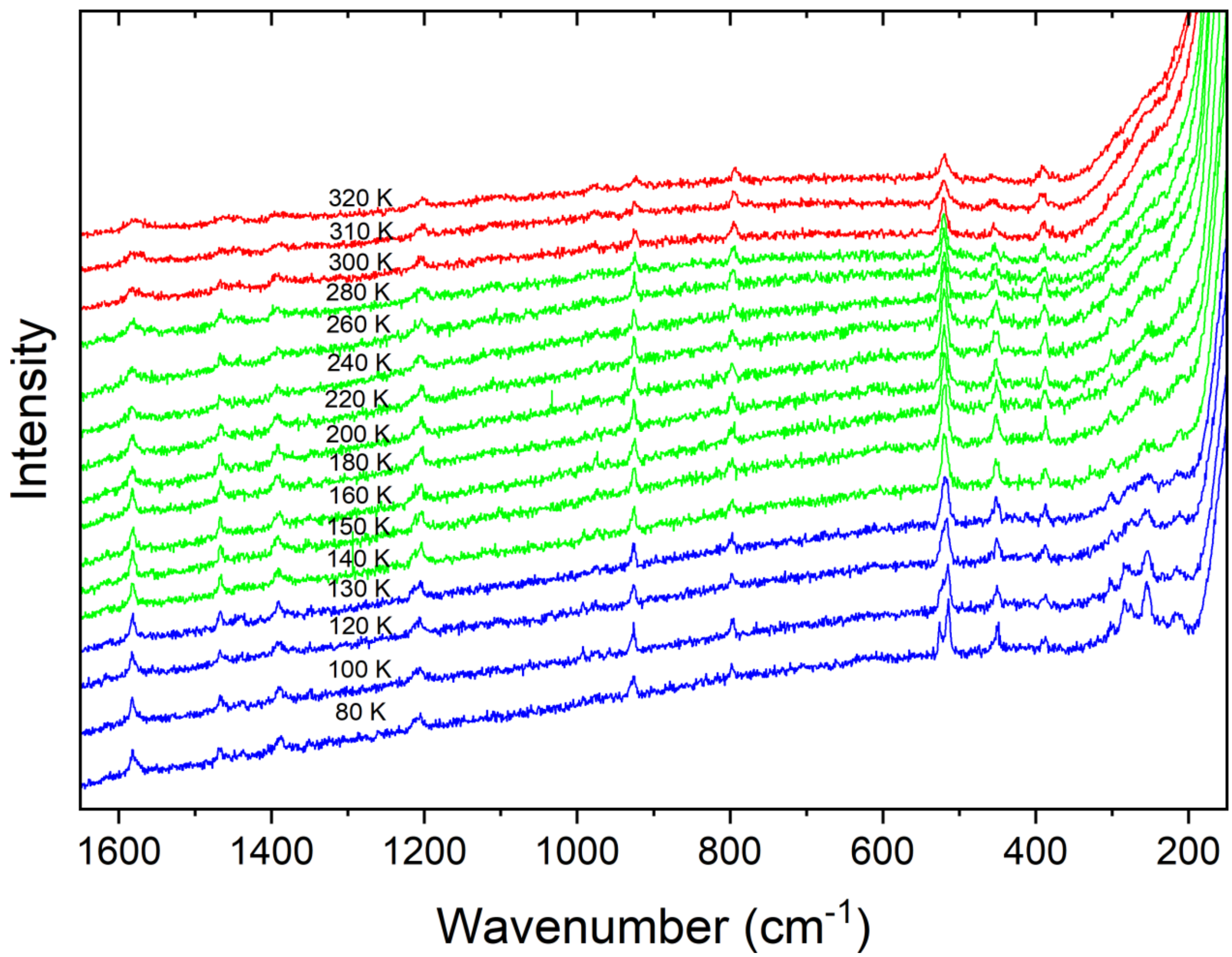

Figure S9. Temperature-dependent Raman spectra of $IPA_2FAPb_2Br_7$ in the 1650-150 cm^{-1} range.

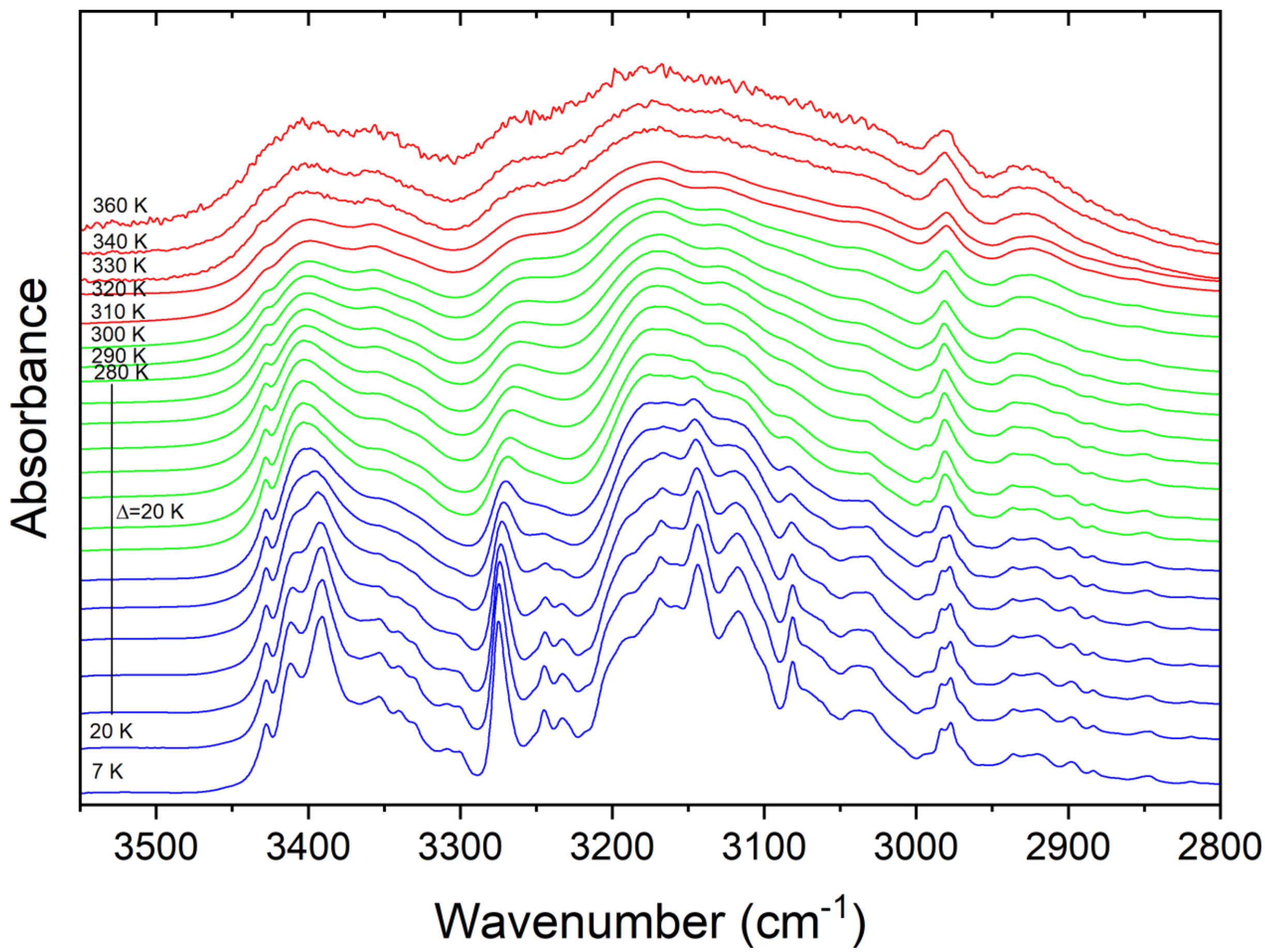

Figure S10. Temperature-dependent IR spectra of $IPA_2FAPb_2Br_7$ in the 3550-2800 cm^{-1} range. Spectra measured at 330, 340 and 360 K were collected using stand-alone IR microscope.

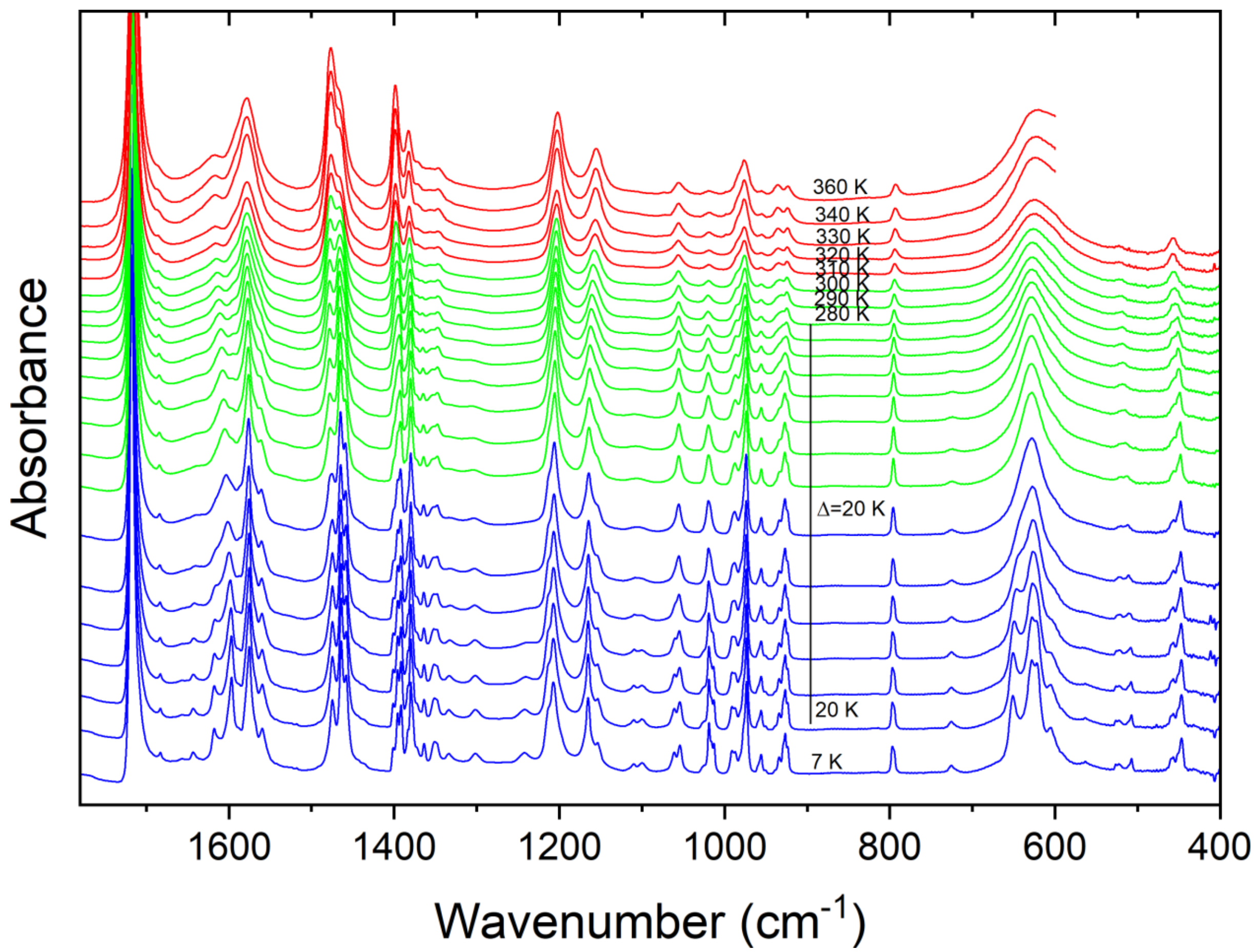

Figure S11. Temperature-dependent IR spectra of $IPA_2FAPb_2Br_7$ in the 1780-400 cm^{-1} range measured using IR cryostat (7-320 K) and in the 1780-600 cm^{-1} range using stand-alone IR microscope (330, 340 and 360 K).

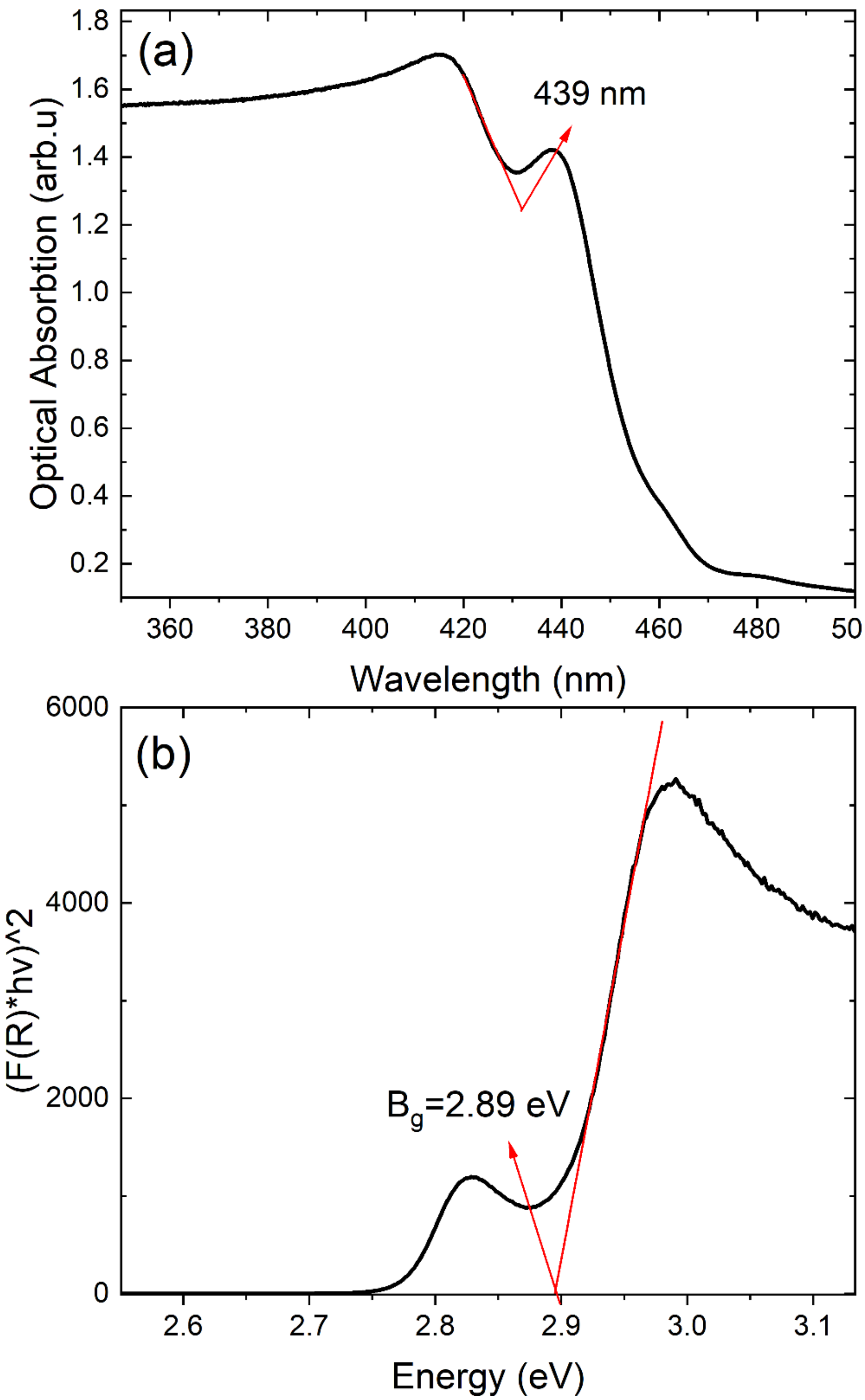

Figure S12. (a) RT diffuse-reflectance spectrum and (b) energy bandgap of $IPA_2FAPb_2Br_7$ estimated by x-axis intersection point of the linear fit of the Tauc plot.

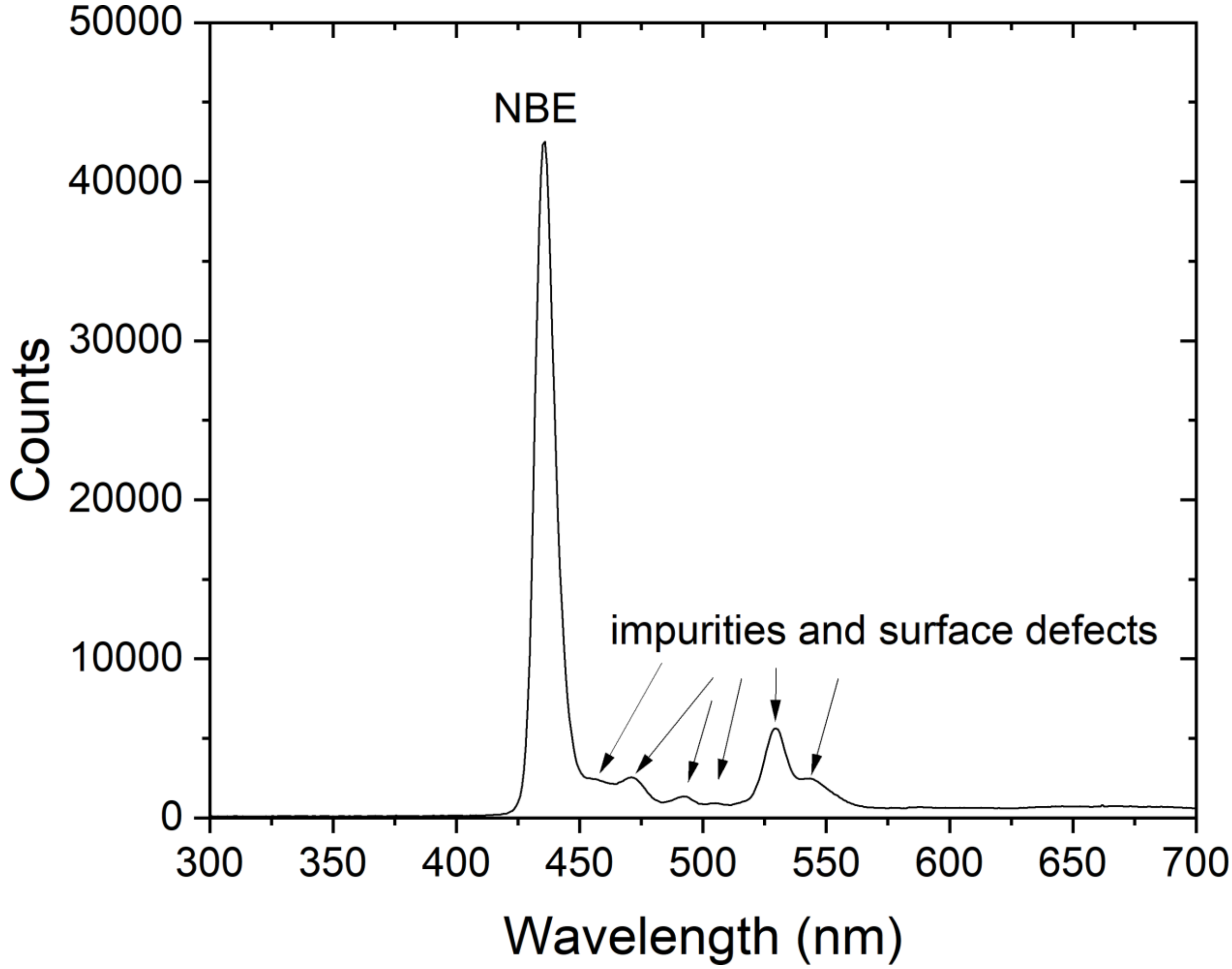

Figure S13. $IPA_2FAPb_2Br_7$ PL spectrum in the 300-700 nm range at 20 K.

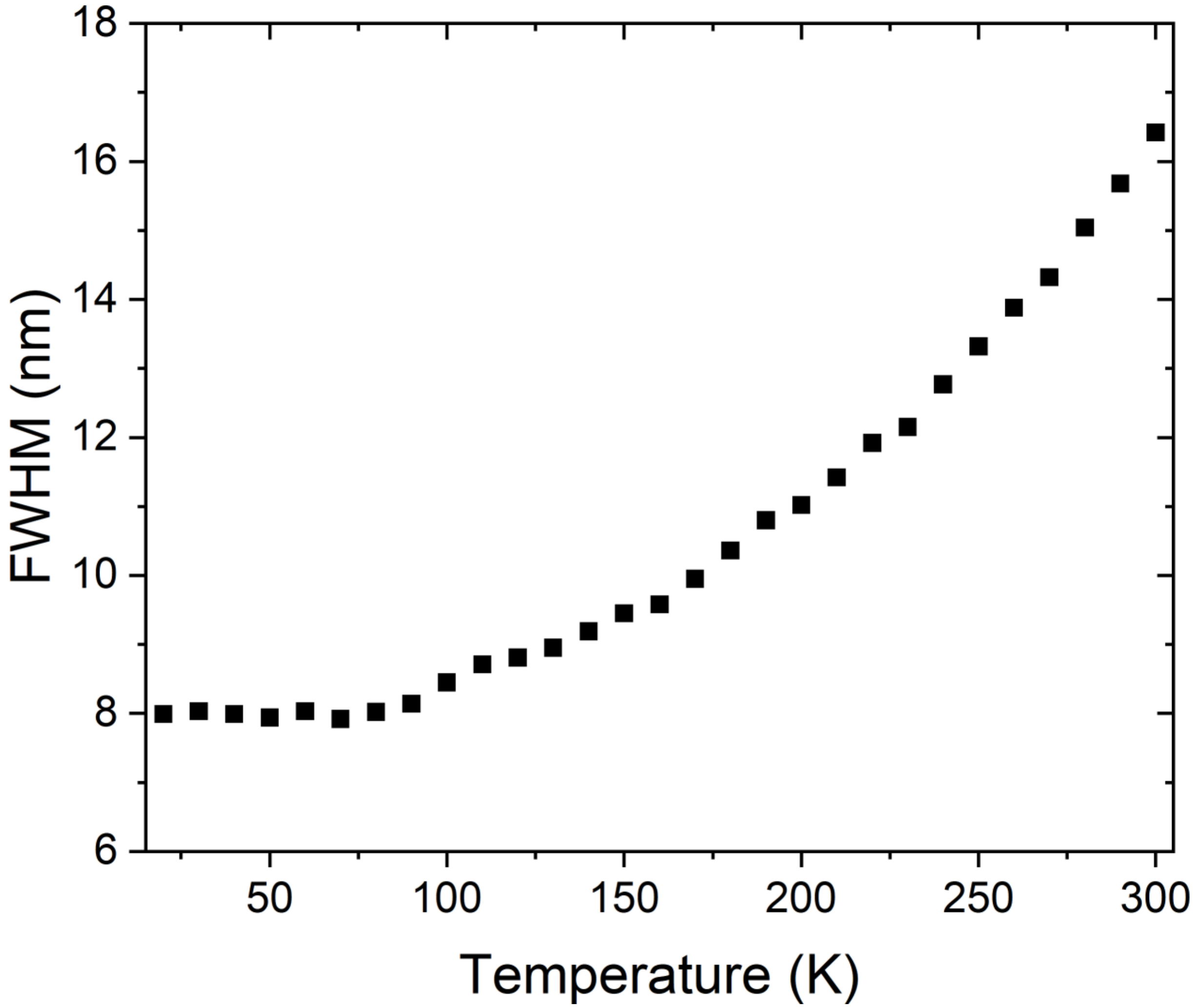

Figure S14. The full width half maximum (FWHM) values for narrow band emission of $IPA_2FAPb_2Br_7$ as a function of temperature.

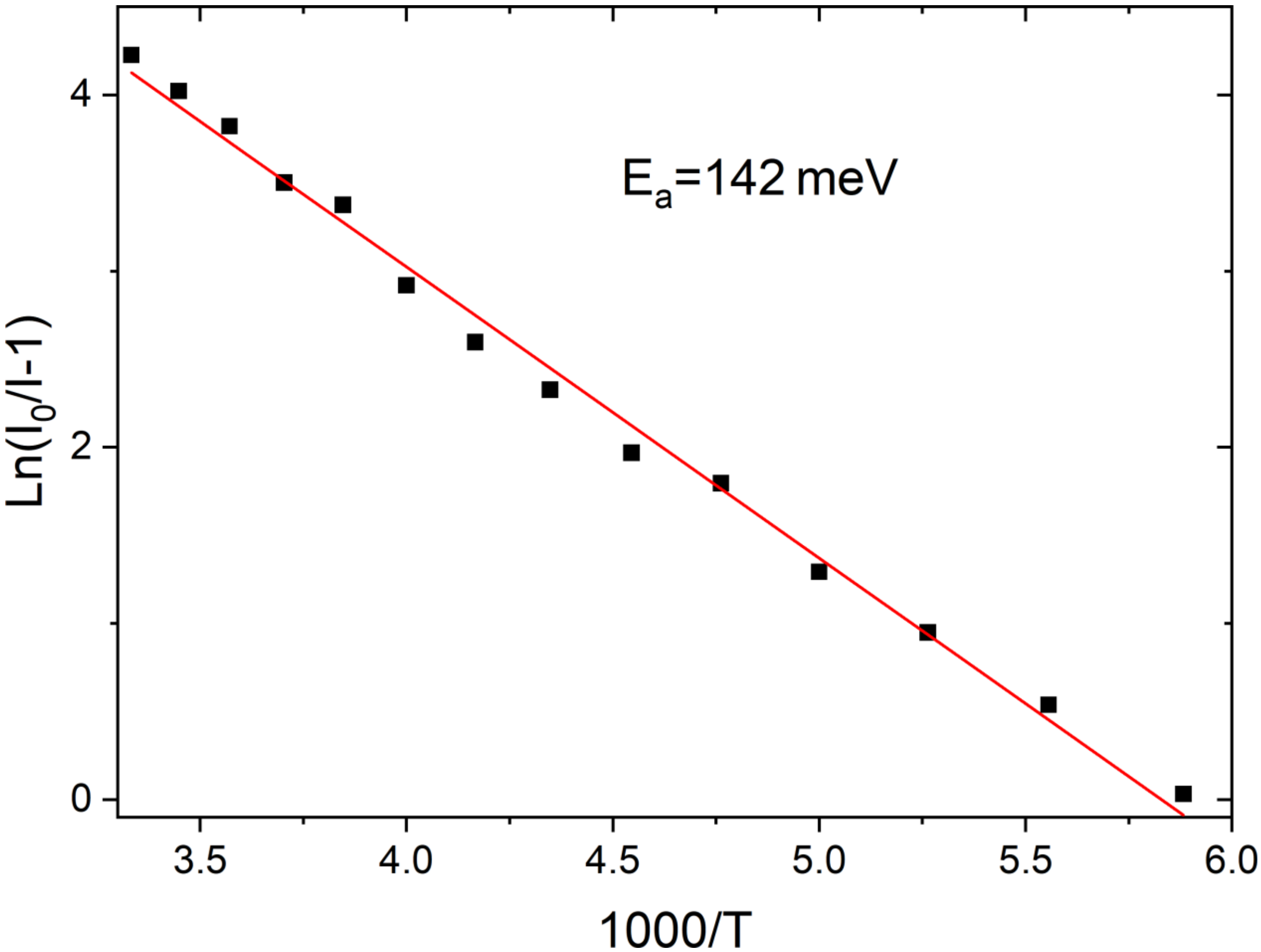

Figure S15. Logarithm of I_0/I-1 as a function of 1/T of LE PL of $IPA_2FAPb_2Br_7$ during heating from 170 to 300 K. E_a was extracted by fitting the Arrhenius equation.
